# Phototoxicity of cyclometallated Ir(III) complexes bearing a thio-bis-benzimidazole ligand, and its monodentate analogue, as potential PDT photosensitisers in cancer cell killing

**DOI:** 10.1007/s00775-023-02031-z

**Published:** 2024-01-06

**Authors:** Marta Martínez-Alonso, Callum G. Jones, James D. Shipp, Dimitri Chekulaev, Helen E. Bryant, Julia A. Weinstein

**Affiliations:** 1 Department of Chemistry, University of Sheffield, Sheffield, S3 7HF UK; 2 Department of Oncology and Metabolism, Medical School, The University of Sheffield, Beech Hill Road, Sheffield, S10 2RX UK

**Keywords:** PDT, Anticancer drugs, Iridium, Phototoxicity

## Abstract

**Abstract:**

Two novel cyclometallated iridium(III) complexes have been prepared with one bidentate or two monodentate imidazole-based ligands, **1** and **2**, respectively. The complexes showed intense emission with long lifetimes of the excited state. Femtosecond transient absorption experiments established the nature of the lowest excited state as ^3^IL state. Singlet oxygen generation with good yields (40% for **1** and 82% for **2**) was established by detecting ^1^O_2_ directly, through its emission at 1270 nm. Photostability studies were also performed to assess the viability of the complexes as photosensitizers (PS) for photodynamic therapy (PDT). Complex **1** was selected as a good candidate to investigate light-activated killing of cells, whilst complex **2** was found to be toxic in the dark and unstable under light. Complex **1** demonstrated high phototoxicity indexes (PI) in the visible region, PI > 250 after irradiation at 405 nm and PI > 150 at 455 nm, in EJ bladder cancer cells.

**Graphical abstract:**

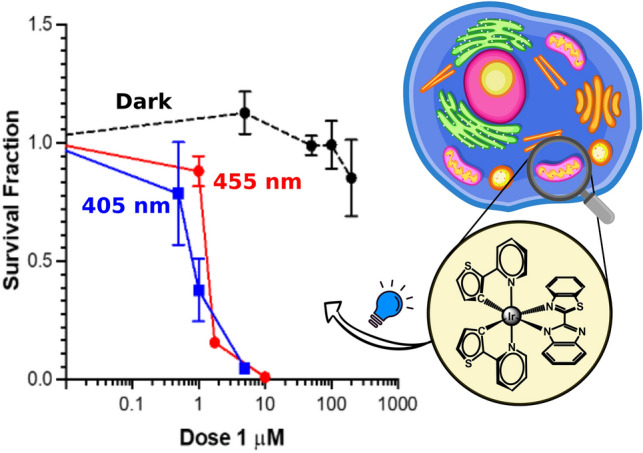

**Supplementary Information:**

The online version contains supplementary material available at 10.1007/s00775-023-02031-z.

## Introduction

Cancer is one of the most malignant diseases in the world. In 2016, it was among the 10 most frequent causes of death, especially in upper-middle and high-income countries [1]. Although new therapies are emerging, surgery and chemotherapy are still the most frequently used techniques to treat cancer. Metal-containing anticancer drugs such as cisplatin and its derivatives are among the best anticancer drugs up to date. Nevertheless, they have lots of side effects in patients because they usually target all proliferative tissues [2]. In this context, Photodynamic Therapy (PDT), widely used to treat actinic keratosis [3, 4], acne [5], age-related macular degeneration [6], barrets oesophagus [7], viral warts [8], periodontal disease [9], or infectious diseases [10], is gaining more and more attention, for its localised effects. PDT requires a photosensitizer (PS), a source of light and molecular oxygen. When a PS is irradiated with light of a specific wavelength, PS molecules absorb light and populate a singlet excited state, which in turn populates a triplet excited state, ^3^PS, in the process called “intersystem crossing”. Afterwards, two pathways of ^3^PS interaction with oxygen are possible. (a) Type I or electron transfer (eT): the ^3^PS transfers an electron to molecular oxygen (^3^O_2_) releasing ROS species. (b) Type II or energy transfer (ET): the ^3^PS transfers its energy to molecular oxygen (^3^O_2_), leading to population of singlet oxygen (^1^O_2_). Both ROS and (^1^O_2_) are reactive species able to kill cells [11]. As ROS (^1^O_2_) are only produced at local sites of irradiation, PDT is more specific to tumour cells than most existing chemotherapies. To this end, many transition metal complexes are being discovered as efficient photosensitisers, owing to their efficient absorption of visible light, followed by ultrafast population of a triplet excited state – facilitated by the presence of a heavy atom, the metal centre – which in turn ensures long excited state lifetime (hundreds of nanoseconds or longer), and the high yields of ROS. For example, a photo-active Ru(II) complex, TLD-1433, prepared by McFarland and co-workers is in phase II clinical trials [12–14] .

Amongst transition metal complexes, many Ir(III) complexes have been reported as promising photosensitizers for PDT [15–18], and reviewed in, for example, [19]. Our previous study reported phototoxic properties of two cyclometallated Ir(III) complexes bearing bisbenzimidazole or its methylated analogue, N-methylated bisbenzimidazole. The complex with bisbenzimidazole, carrying an -NH group, displayed low dark toxicity and a high phototoxicity index (PI) at 405 nm (LD_50dark_ > 100 µM, PI > 333), whereas its N-methylated analogue was highly toxic in the dark and showed low PI (LD_50dark_ = 6.2 µM, PI = 12.4) [20].

Usually, such complexes have a general formula of [Ir(N^C)_2_(NN)], with two cyclometallating, and one bidentate diimine ligand. N-monodentate ligands have not been extensively explored in complexes for PDT. The limited examples reported include imidazole-based Ir(III) complexes as PDT and PACT (photoactivated chemotherapy) agents with PI values up to 61 [21], and imidazole complexes of Pt(II), Ir(III) and Ru(II) in reductive media containing glutathione (GSH)[22] which undergo photo-triggered release of the ligands.

**Fig. 1:**
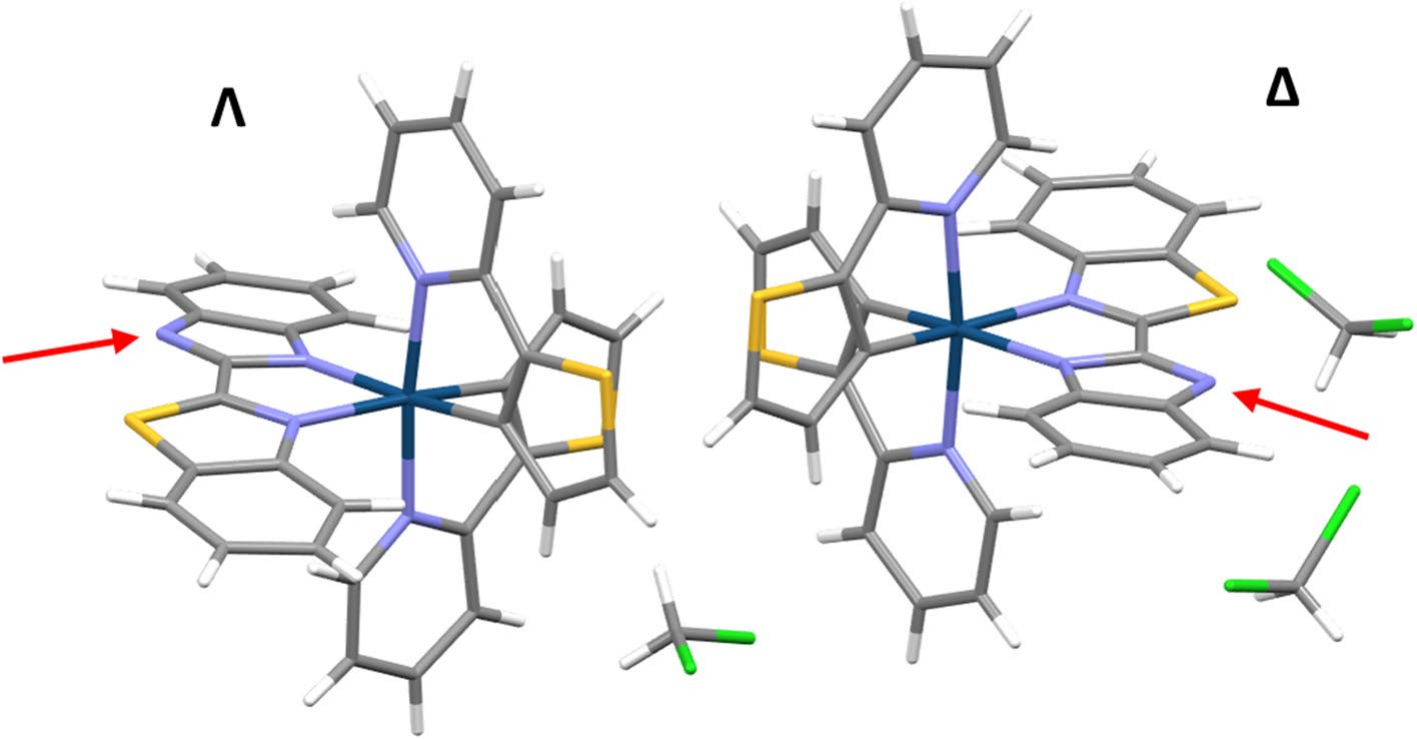
Asymmetric unit of complex **1** showing the optical isomers Λ and Δ, solved by X-ray diffraction. Red arrows indicate the lack of the hydrogen in what would be the NH group

In this work, we present the photodynamic effect of two Ir(III) imidazole-based photosensitizers, one with a chelating diimine ligand and the other with two N-monodentate ligands. The complexes were designed with the aim of shifting the absorption towards the more tissue penetrating red region of the spectrum but maintaining the imidazole -NH functionality, reported to be a relevant scaffold in PDT [20, 23–25]. With this goal in mind, we prepared a sulfur-containing analogue of bis-benzimidazole, 2-(2’-benzothiazolyl)-benzimidazole (btzbimH, Fig. 1), and combined this diimine ligand with 2-(2-thienyl)pyridine (thpy) as the cyclometallating ligand. One of the low-lying excited states in this type of complexes is intraligand charge-transfer state to the pyridine from either phenyl(–) or thienyl(–), components of the N^C ligands. Thpy contains a more electron-donating thienyl group than the phenyl group in the phenyl-pyridine (ppy), leading to complexes bearing thpy possessing a lower-energy ILCT state, shifting absorption maximum to the more favourable for the PDT red region of the spectrum [26–28]. Since the -NH moiety on the ancillary ligand(s) could modulate the total charge of the complex, through the acid–base equilibrium, affecting the cellular uptake and localization of the complex [23, 25, 29], the effect of pH on photophysical properties of the complexes was investigated. The photophysical properties, chemical and photochemical stability, and the propensity of the complexes to act as photosensitisers were investigated and the influence of the monodentate vs. bidentate N^N ligands established. Furthermore, complex [Ir(thpy)_2_(btzbim)]^+^ (**1)** was shown to be a promising PDT agent in EJ cells (bladder cancer cells) under both 405 nm and 455 nm irradiation, with appreciable phototoxicity indices.

## Results and discussion

### Synthesis of the complexes

**Scheme 1.**
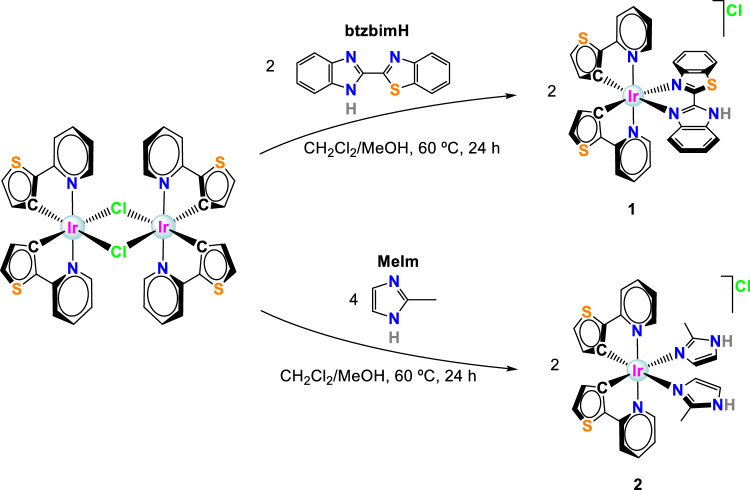
Synthetic scheme of complexes **1** and **2**. Note: All the compounds were prepared as the racemic mixtures of Δ and Λ isomers. The drawings show only one of them for illustrative purposes. Likewise, only one variant of the stereochemistry is shown for the dimeric complex

The starting dimer [Ir(*µ*-Cl)_2_(thpy)_2_], was prepared following the Nonoyama protocol [30]. IrCl_3_·*x*H_2_O was reacted with the ligand 2-(2-Thienyl)pyridine (thpy) to yield a chloride-bridge binuclear complex. Then, the dimer was refluxed with the corresponding ligands in a dichloromethane/methanol mixture for 24 h to yield complexes **1** and **2** (Scheme 1 and Experimental section) as the racemic mixture.

The ligand 2-(2′-benzothiazolyl)-benzimidazole (bztbimH) was synthesized according to a literature procedure[31] using a microwave reactor (see Experimental section).

A complete characterization was performed by NMR (1D ^1^H- and ^13^C-NMR, and 2D NMR methods, COSY and HSQC, Figure S1, S2, S4, S5) and ESI–MS/TOFMS (Figs. S3, S6). As expected, the ^1^H NMR spectrum of complex **1** showed asymmetry, whereas the NMR spectrum of complex **2** exhibited a symmetric complex. Complex **2** slightly decomposed during the recording of the NMR (Fig. S4), resulting in the appearance of a set of peaks corresponding to an asymmetric product. In order to explain this phenomenon, the lability of the monodentate ligands as well as the stability of the complexes have been investigated and are described below. The MS spectra displayed the typical isotopic distribution pattern of Iridium complexes. Interestingly, the MS spectrum of **2** showed three peaks, assigned to the exact mass main peak [M]^+^, the loss of one of the monodentate MeIm ligands [M-MeIm]^+^, and the replacement of the MeIm ligand by a molecule of acetonitrile (used as the solvent) [M-MeIm + CH_3_CN]^+^.

The single crystal X-ray structure of **1** was determined, using single crystals formed by slow evaporation of the solvent from solution of **1** in dichloromethane (Fig. 1). The asymmetric unit contains two complexes corresponding to the two enantiomers, Λ and Δ, and three CH_2_Cl_2_ molecules. The structure shows off-centred π-π stacking interactions between the btzbim ligands. The connection between both isomers is reinforced by CH-π interactions (see Fig. S7; Table S1–S4), also involving the thienyl ring. Unexpectedly, the complex was found to be neutral, since neither a counterion nor electronic density in the nitrogen of the imidazole ring (see red arrows in Fig. 1) was found. Accordingly, the ^1^H NMR spectrum of **1** does not show any peak for the NH. Another evidence of the lack of the H-atom on the imidazole is that, in both isomers, the N atom is participating in weak hydrogen bond interactions as an acceptor (C–H···N, see Table S5)[32]: in the Λ isomer, H-bonding occurs with a CH_2_Cl_2_ molecule, and in the Δ isomer, with a hydrogen atom of the aromatic ring of another Δ isomer.

The complex presents a distorted octahedral geometry, with the N-donor atoms of the two cyclometallated ligands being in *trans* position to one another. The Ir-C and Ir-N bond lengths are in the usual range reported for similar complexes [27, 33–35], with the Ir-N distances (2.02–2.07 Å) in the thpy ligand longer than Ir-C ones (1.96–2.02 Å), due to the *trans* effect. The bite angles for the cyclometallating ligands are smaller than 90°, evidencing the distorted geometry. Moreover, for the btzbim ligand, the bite angles (75° and 77°) are considerably smaller than for the cyclometallated ones (79–80.5°)[33, 36, 37] The btzbim ligand presents a twisted non-planar geometry, with angles between the planes of the benzimidazole and benzothiazole being 12.68° for the Λ isomer and 7.29° for the Δ isomer.

### Photophysical properties

**Fig. 2:**
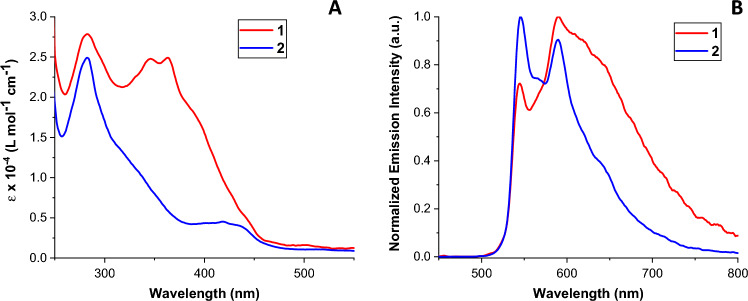
**A**UV–Vis absorption and **B** normalized emission intensity spectra of complexes** 1** (10^–5^ M, λ_exc_ = 440 nm) and **2** (2 × 10^–5^ M, λ_exc_ = 432 nm) under argon in CH_2_Cl_2_ at r.t

The photophysical properties of the complexes in CH_2_Cl_2_ were investigated under air and under argon atmosphere (Figs. S8 and S9). Both **1** and **2** exhibit strong absorption bands at 250–350 nm, due to spin-allowed ^1^π–π* ^1^LC transitions. The low energy bands, 350–450 nm, correspond to spin-allowed ^1^MLCT, ^1^LLCT transitions, whereas the weak absorption tails above 450 nm would comprise spin-forbidden ^3^MLCT, ^3^LLCT and ^3^π–π* LC transitions (Fig. 2A). Upon excitation of solution of **1** or **2** at room temperature, the orange-red emission was observed (Fig. 2B). The spectra have pronounced vibronic structure with the two main peaks being the same for both complexes **1** (546 and 593 nm) and **2** (546 and 590 nm), indicating emission with a considerable ^3^LC character. In addition, the spectrum of **2** (including the shoulder at 640 nm) has the same shape to that reported for [Ir(thpy)_2_(1,1′-dimethyl-2,2’-biimidazole)]PF_6_.[34] These observations point out a main contribution from a ^3^LC transition involving the thpy ligands, for **2**, whereas for **1** an additional contribution of emission from a CT state may play a role. The emission quantum yields (measured in air) for **1** and **2** in CH_2_Cl_2_ (0.029 and 0.016, respectively) are relatively small, and the characteristic emission lifetime is approximately 200 ns for both complexes. The excited state lifetimes in deoxygenated solution are considerably longer than in aerated solutions, with *ca.* 2 µs for **2**, and a somewhat shorter, ca. 800 ns, for **1**, which could also be due to a mixed CT/LC nature of the emissive state in **1**. Table 1 gathers the photophysical properties of complexes **1** and **2** under air and argon.

**Table 1:** Photophysical properties of **1** (10^–5^ M) and **2** (2 × 10^–5^ M) in CH_2_Cl_2_

Complex	λ_exc_ [nm] (ε [M^−1^ cm^−1^])	λ_em_ [nm]^a^	Φ_em_^b^	Φ_s_ (^1^O_2_)^c^	τ [ns]	k_r_ [× 10^–5^ s^−1^]	k_nr_ [× 10^–5^ s^−1^]
1-Air	279 (27,778), 348 (23,795), 362 (23,727), 394 (sh, 18,282), 440 (5527)	λ_440_ 544, 593	0.029 ± 0.002	0.40	230	1.3 ± 0.1	45 ± 1
1-Ar	283 (27,851), 346 (24,768), 363 (24,919), 391 (sh, 17,759), 440 (9487)	λ_440_ 545, 590	–	–	800 (20%), 300 (80%)^d^	–	–
2-Air	282 (23,124), 419 (4753), 512 (1275)	λ_432_ 546, 591	0.016 ± 0.001	0.82	196	0.82 ± 0.05	50 ± 1
2-Ar	283 (24,890), 418 (4528), 512 (1089)	λ_432_ 546, 590	–	–	1980	–	–

^a^Excitation wavelength as stated

^b^Relative to [Ru(bpy)_3_]^2+^ Φ_em_ in CH_2_Cl_2_ (0.029 [38])

^c^Relative to perinaphthenone (Φ_s_ = 1)

^d^We note the biexponential decay of emission of **1** under Ar atmosphere. It is possible that there is an emission from two triplet states, a primarily ILCT one, and a primarily MLCT one. There was no obvious concentration dependence. The excitation spectra recorded at different emission wavelengths follow the absorption spectra (see SI) hence emission originates from the parent compound

### Stability and photostability of the complexes

**Fig. 3:**
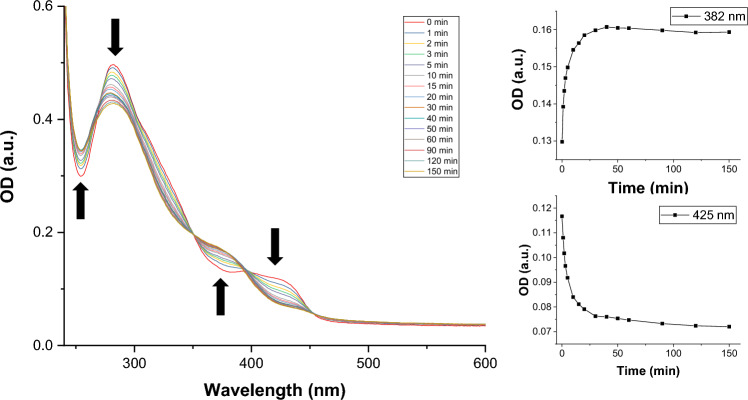
Time-dependent UV–vis spectra of complex **2** in water (2% DMSO) after irradiation (405 nm, 20 mW). Inset: kinetic traces recorded at 382 nm (growth of absorbance of the product) and 425 nm (prevailing absorbance by starting material)

**Scheme 2.**
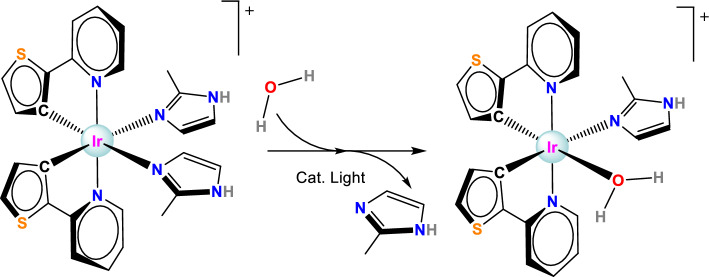
Proposed scheme for the photo-uncaging of the imidazole ligand and its replacement with a water molecule in complex **2**

The photostability of the complexes was tested in water (1 or 2% DMSO), under irradiation with a 405 nm diode (20 mW) with the process followed by UV–vis absorption spectroscopy. Complex **1** was stable after 90 min of irradiation (Fig. S10), the slight changes observed in the spectra were possibly due to precipitation. However, the absorption spectrum of complex **2** showed pronounced changes upon irradiation, with two isosbestic points (Fig. 3). Moreover, the emission of **2** completely disappeared after 150 min of irradiation with 405 nm light (Figure S11), suggesting the release of some of the ligands, as reported by *Wu *et al.[21] The same changes, although occurring slower, were also detected in a sample exposed to ambient light (Figure S12). A time-dependent ^1^H-NMR experiment was conducted in DMSO-d_6_/D_2_O (70/30) to try to work out the molecular changes (Figs. S13–S14). Unexpectedly, the changes in the NMR spectra in this solvent mixture occur much slower than in water (monitored by UV–vis spectroscopy). The NMR data confirm that one of the MeIm monodentate ligands was released and that the main species responsible for the changes were water molecules. Thus, we propose a substitution mechanism (see Scheme 2), in which the imidazole is replaced by a water (or another solvent) molecule.

In order to check the effect of light, we performed the same experiment for **2** in dark conditions in water, PBS and in clear cell medium. Figure S15 displays the effect of the different solvents on the absorption spectra, with large changes observed in aqueous solution, but negligible changes occurring in cell media. Thus, complex **2** is not stable in water or PBS, but it is stable in cell media under irradiation.

### Protonation/deprotonation process

The pK_a_ for **1** was 6.97 in a PBS buffered solution (Figure S16). This means complex **1** will be mainly present in the deprotonated, neutral form in the cell culture, whose pH is 7.4. The protonated, charged form will dominate in acidic environments, such as tumour cells (pH 6.5–6.8) or lysosomes (pH 4.5–5.5) [23]. For a similar complex [Ir(ppy)_2_(pybz)] (where ppy = 2-(2-phenylpyridine) and pybz = 2-(2-pyridyl)benzimidazole), the emission spectra of deprotonated and protonated forms in solution are different. However, the emission emanated from cells incubated with either form for 5 min (for [Ir(ppy)_2_(pybz)], pK_a_ = 6.6, intracellular pH ~ 7.4), indicating that the uptake was not affected by charge and (de)protonation [39], and that only one form (deprotonated) is present inside the cells.

**Scheme 3.**
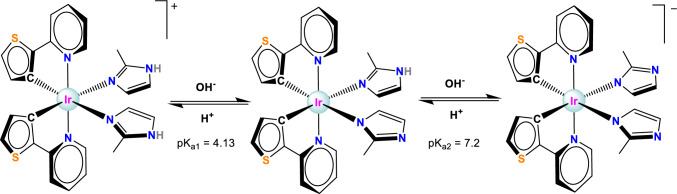
Protonation/deprotonation process scheme of complex **2**

For complex **2**, the accurate measurement of pK_a_ was problematic due to its instability. However, a fast measurement allowed us to estimate two pK_a_ values: pK_a1_ ~ 4.13 and pK_a2_ ~ 7.20 (Scheme 3 and S17), each of these values were lower than the one for the free 2-methylimidazole (pK_a_ = 7.86 [40]), as can be expected due to coordination to the metal. If one assigns the two pK_a_ values to the consecutive deprotonation of the NH of the two ligands, one can correlate the decomposition of **2** with the pH. In distilled water with pH 5.8, one of the imidazoles would be in the deprotonated form, whereas the other one would be protonated and prone to uncaging. In cell media at pH 7.4, both imidazoles would be deprotonated and it will probably be more difficult to release the ligand. This hypothesis could also explain the observed slower uncaging process in the NMR experiments performed in D_2_O which is less acidic than H_2_O.

### Singlet oxygen sensitization

The decrease in the emission intensity and lifetime in presence of oxygen pointed out to molecular oxygen as a quencher of the emissive state, which typically result in production of singlet oxygen. The yield of singlet oxygen production in CH_3_CN solutions was determined by direct detection of its emission at 1270 nm, using perinaphthenone as the reference (Φ_s_ = 1 in CH_3_CN [41]). Singlet oxygen quantum yields were high, 0.40 for **1** and 0.82 for **2**, with the lower value for **1** correlating well with its shorter excited state lifetime, and a smaller estimated rate constant of quenching by oxygen (Table 1).

### Cyclic voltammetry

**Fig. 4:**
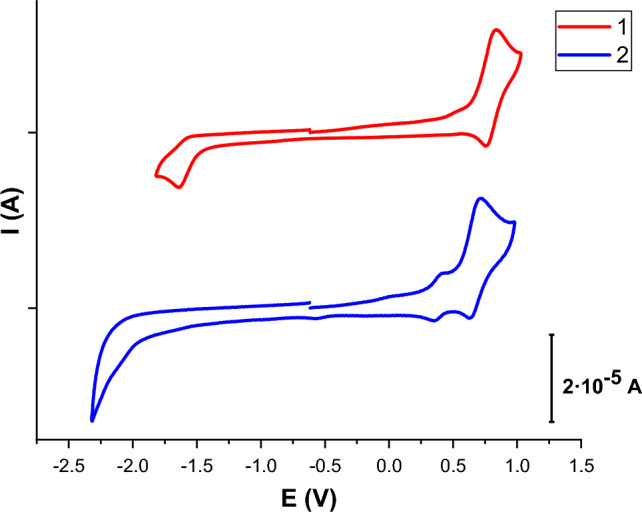
Cyclic voltammograms of complexes **1** and **2** in CH_2_Cl_2_ solution (10^−3^ M), using 0.4 M [NBu_4_][PF_6_] as supporting electrolyte. Scan rate: 100 mV/s. Potential vs. Fc/Fc^+^

The electrochemical behaviour of **1** and **2** was investigated by cyclic voltammetry in CH_2_Cl_2_ (Fig. 4 and Table 2 with a glassy carbon working electrode, a platinum wire counter electrode and Ag/AgCl (0.1 mol dm^−3^) reference electrode. The redox couple Fc/Fc^+^ was used as the internal reference. The main oxidation peaks at + 0.80 V (**1**) and + 0.67 V (**2**) are assigned to the oxidation of the Ir-thpy environment (Ir^III^ to Ir^IV^). The more positive value for **1** is the consequence of the stronger LFSE (ligand field stabilization energy) for the bidentate chelate ligand than for the two monodentate ligands in **2**. The additional, small, reversible oxidation process observed for **2** at + 0.39 V corresponds to the oxidation of the Cl^−^ anion. The lack of the same oxidation peak in **1**, also confirms the absence of a chloride counterion in the complex and indirectly, its neutral nature. The reduction processes (− 1.61 V for **1** and − 2.10 V for **2**) are irreversible and they are usually ascribed to the ancillary ligands. The more negative value for **2** might be caused by the introduction of electron-donating methyl groups.

**Table 2:** Electrochemical properties (vs. Fc/Fc^+^) of **1** and **2** in CH_2_Cl_2_ solution (10^−3^ M), using 0.4 M [NBu_4_][PF_6_] as supporting electrolyte. Scan rate: 100 mV/s

Compound	E^ox^_1/2_	E^red^_1/2_	ΔE_1/2_
1	+ 0.80	− 1.61	2.41
2	+ 0.39, + 0.67	− 2.10	2.77

### Transient absorption spectroscopy

**Fig. 5:**
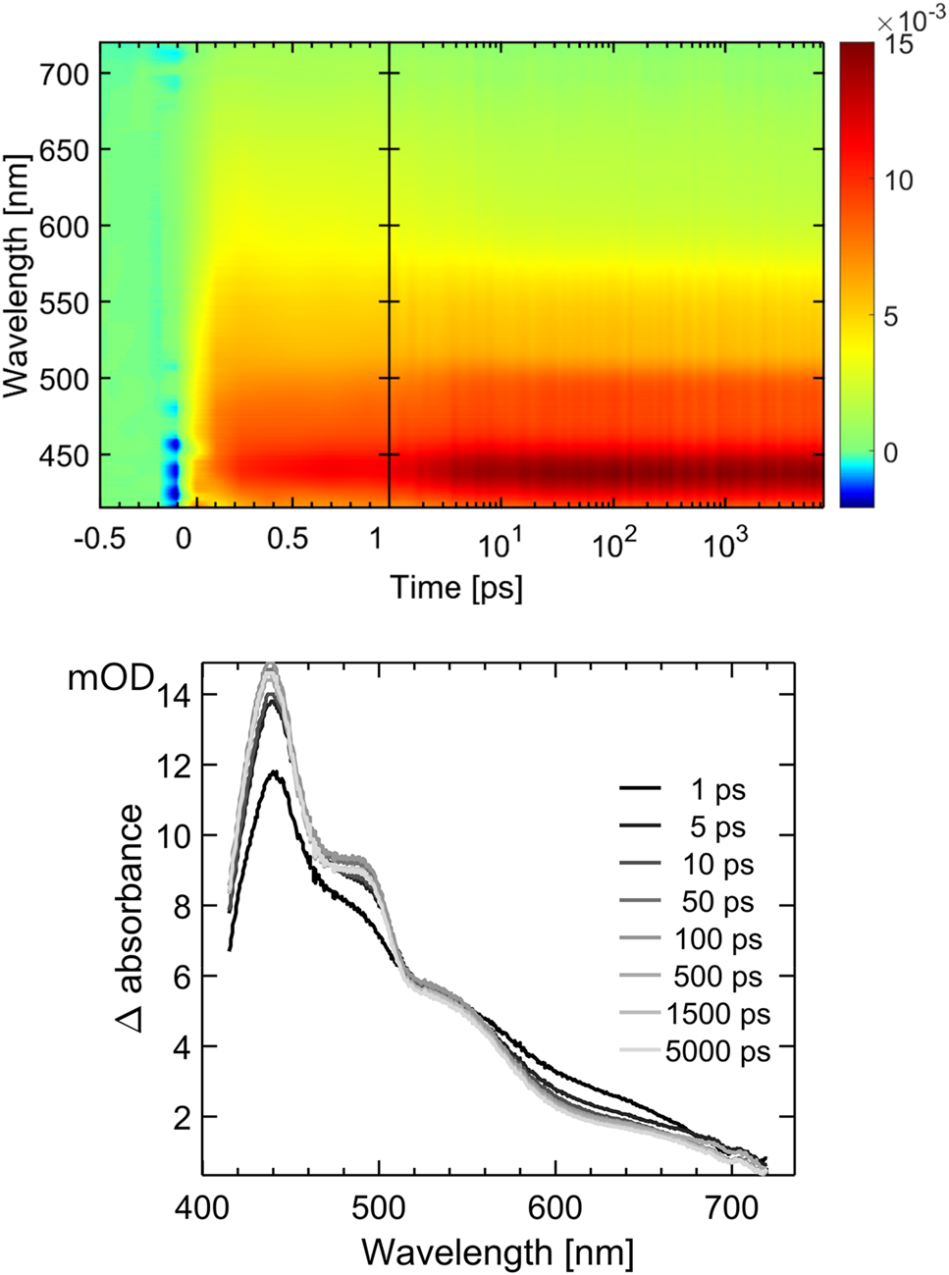
Transient absorption data for **1** in aerated DCM, excitation 400 nm, 40 fs. Top: 2D-representation of the complete dataset; bottom—spectra at selected time delays after the excitation

The transient absorption spectra obtained after 400 nm, 40-fs excitation of the solution of **1** in DCM at r.t., on the time scale 0–8 ns, are shown in Fig. 5. The data were fitted using Glotaran global analysis routine with a sequential 3-exponential model, with the time-constants of 1.32 ps, 14 ps, and an additional “infinity” value to account for the final, emissive state (the latter was arbitrary fixed as 225 ns, to match emission lifetime). The spectra associated with each time constant are shown in the SI (decay-associated spectra). The transient absorption spectra at early times show slight vibrational progression (455 nm, 495 nm, and 550 nm) typical of intraligand excited states. These features become more pronounced, with the time-constant of 1.32 ps, whilst a lower-energy broad absorbance at ca. 640 nm (where one might expect the diimine radical-anion absorbance) decreases in intensity. Further sharpening of the spectral features occur with 14 ps timeconstant (perhaps due to vibrational cooling), after which the spectral shape remains unchanged on the timescale of the experiment (8 ns).

### Cytotoxicity and phototoxicity

**Fig. 6:**
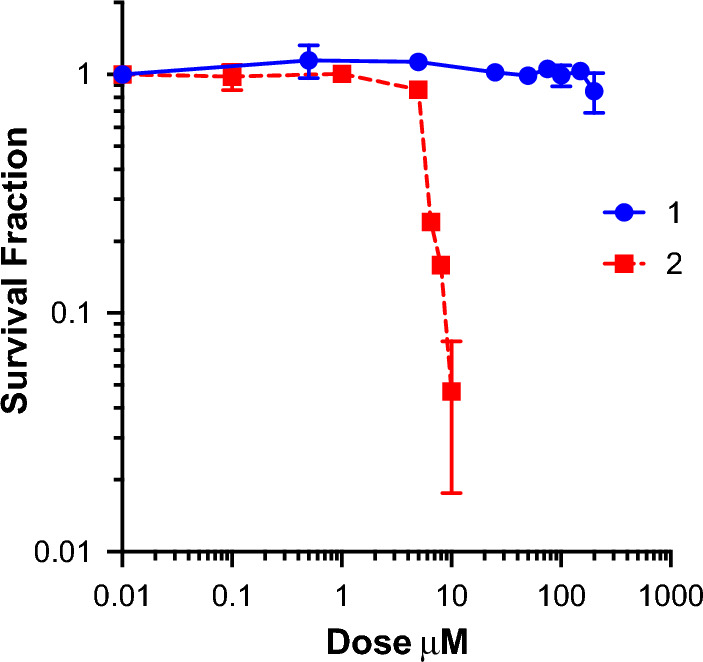
Dark toxicity of **1** and **2** in EJ cells after 2 h incubation. Data are normalised to DMSO alone control, mean and SEM of 3 independent repeats each performed in triplicate is shown

**Fig. 7:**
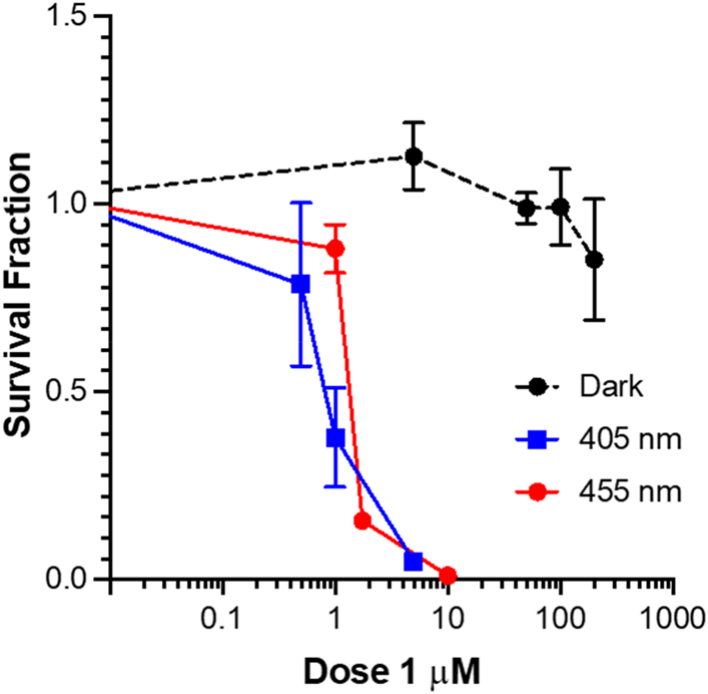
Dark and light (λ_exc_ = 405 nm, 20 mW/cm^2^, 3 min and λ_exc_ = 455 nm, 30 mW/cm^2^, 3 min) toxicity of **1.** Data are normalised to DMSO alone control, mean and SEM of 3 independent repeats each performed in triplicate is shown

The cytotoxicity of the complexes in the dark and upon light irradiation was evaluated in bladder cancer EJ cells after 2 h incubation. Compound **2** was found to be cytotoxic in the dark (LD_50_ = 5.77 µM), whereas **1** was not cytotoxic within the limits of testing (> 200 µM, solubility limit) (Fig. 6, Table 3). Therefore, **1** was selected for further studies. After 2 h of incubation, cells were irradiated at 405 nm or 455 nm for 3 min and survival analysed by clonogenic assay. At 405 nm the LD_50_ value for **1** was 0.79, resulting in a high PI value > 250 (Fig. 7, Table 3) compared to other Ir(III) similar biscyclometallated complexes [42, 43]. When irradiated at 455 nm, the LD_50_ and PI values decreased (LD_50_ = 1.33 PI > 150,) according to the lower absorption at higher wavelengths. The high toxicity of **2** in the dark is consistent with the high dark toxicity observed by Gao and co-workers for a similar complex. This could be explained as the effect caused by rapid replacement of one of the imidazoles by a glutathione molecule in cellular media [22].

**Table 3:** Cytotoxicity and phototoxicity of **1** and **2** in EJ cells after 2 h

Compound	Dark	405 nm		455 nm	
	LD_50_ dark (µM)	LD_50_ light (µM)	PI	LD_50_ light (µM)	PI
1	> 200	0.79	> 250	1.33	> 150
2	5.77	–	–	–	–

### Cellular uptake and localization

**Fig. 8:**
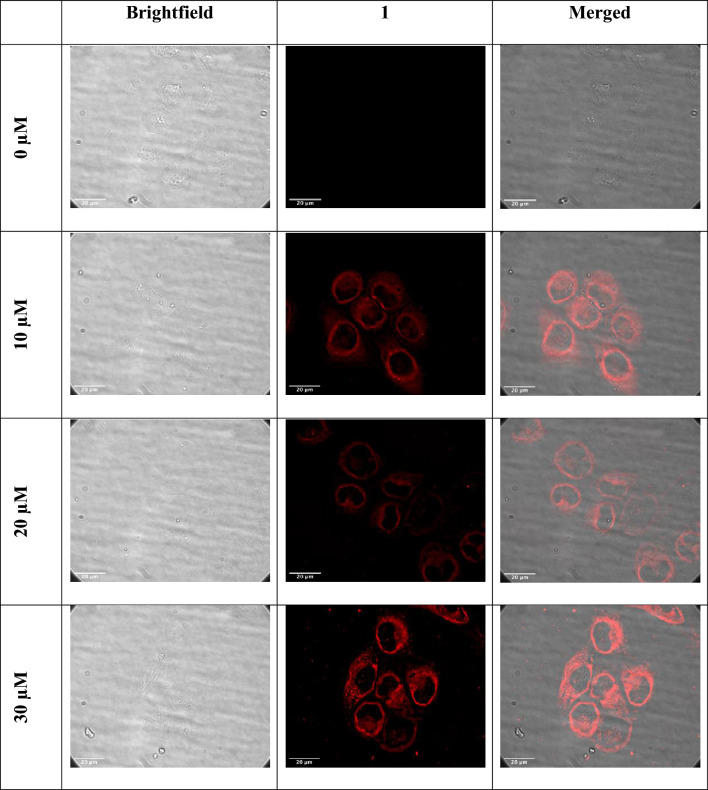
Cellular internalization of complex **1** in EJ cells at different concentrations (0, 10 and 30 μM) after 2 h

The strong emission of the Ir(III) complexes offers a useful tool for imaging, allowing one to follow the uptake process and determine intra-cellular localization of the complexes, which can provide insights into the cell death mechanism. To investigate cellular uptake, dose and time-dependent imaging experiments were performed in EJ cells. For the dose experiment, cells were exposed to increasing concentrations of **1** (0, 10, 30 µM) for 2 h. Cytoplasmic localisation could be clearly seen in Fig. 8 at 10 µM, the intensity of emission (and hence the uptake) was seen to increase when the cells were incubated with a 30 µM solution of **1**.

**Fig. 9:**
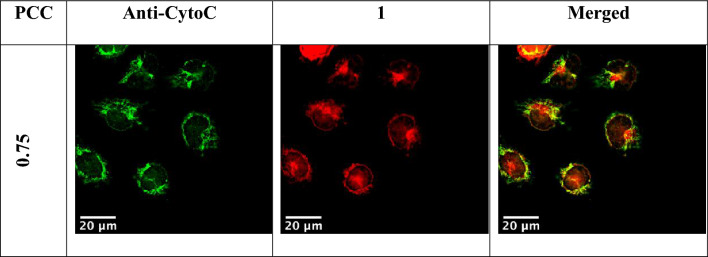
Sub-cellular localisation of complex 1 in EJ cells after 2 h incubation

Initial observations of the sub-cellular localisation of **1** suggest a mitochondrial like location. Co-localisation experiments were therefore performed in EJ cells using immunofluorescent staining for cytochrome c oxidase and utilising the phosphorescence of **1** (Fig. 9). The Pearson’s correlation coefficient (PCC) displays a moderate correlation with a mean value of 0.75. The internalization of **1** in mitochondria could explain the toxicity of the compound after light activation, especially as mitochondria are crucial regulators of the apoptotic pathway.[44, 45]. However, further investigation is needed to examine the location of the portion of 1 that does not-colocalise with mitochondria.

## Experimental part

### Synthesis of the complexes

**[Ir(thpy)**_**2**_**(btzbim)]Cl (1)**. In a 100 mL round-bottomed flask, the precursor [Ir(*µ*-Cl)(thpy)_2_] (39.4 mg, 0.036 mmol) along with the ligand 2-(2’-benzothiazolyl)benzimidazole (19.2 mg, 0.076 mmol) were dissolved in a methanol/dichloromethane mixture (1:1, 10 mL) under argon. The orange mixture was refluxed for 24 h. After this time, the yellowish clear solution was concentrated, and hexane was added to precipitate a yellow solid. The suspension was cooled down in the fridge for 30 min to help in the precipitation process. The crude solid was filtered off and washed with hexane twice. The yellow solid was dried under vacuum. Yield: 24 mg (0.032 mmol, 44%). ^1^H NMR (400 MHz, Chloroform-*d*, 25 °C) δ 7.90 (d, *J* = 7.9 Hz, 1H), 7.78 (d, *J* = 8.2 Hz, 1H), 7.63 (d, *J* = 5.6 Hz, 1H), 7.52–7.41 (m, 6H), 7.39 (d, *J* = 4.6 Hz, 1H), 7.35 (t, *J* = 7.7 Hz, 1H), 7.20 (t, *J* = 7.8 Hz, 1H), 7.12 (t, *J* = 7.5 Hz, 1H), 6.89 (t, *J* = 7.6 Hz, 1H), 6.79 (d, *J* = 8.3 Hz, 1H), 6.63–6.54 (m, 2H), 6.48 (d, *J* = 4.8 Hz, 1H), 6.35 (d, *J* = 4.8 Hz, 1H), 6.13 (d, *J* = 8.3 Hz, 1H) ppm. ^13^C NMR (101 MHz, Chloroform-*d*, 25 °C) δ 165.7 (s, 1C), 165.1 (s, 1C), 164.7 (s, 1C), 164.1 (s, 1C), 150.4 (s, 1C), 137.5 (s, 2C), 137.2 (s, 1C), 132.4 (s, 1C), 131.5 (s, 1C), 129.3 (s, 1C), 128.5 (s, 1C), 127.6 (s, 1C), 125.9 (s, 1C), 125.8 (s, 1C), 122.9 (s, 1C), 122.8 (s, 1C), 121.2 (s, 1C), 119.6 (s, 2C), 117.7 (s, 1C), 117.4 (s, 1C), 116.0 (s, 1C) ppm. ESI + (m/z) = 764.1 [M + H]^+^.

**[Ir(thpy)**_**2**_**(MeIm)**_**2**_**]Cl (2)**. In a 100 mL round-bottomed flask, the precursor [Ir(*µ*-Cl)(thpy)_2_] (100 mg, 0.091 mmol) along with the ligand 2-methyl imidazole (68 mg, 0.83 mmol) were dissolved in a methanol/dichloromethane mixture (1:1, 20 mL) under argon. The orange slurry mixture was refluxed for 24 h. After this time, the orangish clear solution was concentrated and diethyl ether was added to precipitate a yellow solid. The suspension was cooled down in the fridge overnight to help in the precipitation process. The crude solid was filtered off and washed with hexane twice. The yellow solid was dried under vacuum. Yield: 75 mg (0.105 mmol, 58%). ^1^H NMR (400 MHz, DMSO-*d*_6_, 25 °C) δ 12.49 (bs, 2H, H^NH^), 8.69 (bs, 2H, H^9^), 7.81 (t, J = 7.5 Hz, 2H, H^7^), 7.59 (d, *J* = 8.2 Hz, 2H, H^6^), 7.36 (d, *J* = 4.7 Hz, 2H, H^2^), 7.16 (ddd, *J* = 7.4, 5.8, 1.5 Hz, 2H, H^8^), 7.07 (t, *J* = 1.9 Hz, 2H, H^b^), 6.64 (bs, 2H, H^a^), 6.08 (d, *J* = 4.7 Hz, 2H, H^3^), 1.93 (s, 6H, H^d^) ppm. ^13^C NMR (101 MHz, DMSO-*d*_6_, 25 °C) δ 189.1 (s, 2C, C^1^), 163.9 (s, 2C), 151.6 (s, 2C), 138.8 (s, 2C, C^7^), 135.4 (s, 2C), 128.7 (s, 2C, C^2^), 119.8 (s, 2C), 117.4 (s, 2C, C^6^), 116.9 (s, 2C, C^b^), 13.8 (s, 2C, C^d^) ppm. ESI + (m/z) = 595 [M-MeIm]^+^, 636 [M-MeIm + CH_3_CN]^+^, 677 [M]^+^.

## Materials and methods

IrCl_3_·xH_2_O was purchased from Johnson&Mattey and used as received. 2-(2-Thienyl)pyridine and 2-Methylimidazole were purchased from Sigma-Aldrich. Solvents were distilled and dried prior use. All the reactions were carried out under a dry and oxygen free argon atmosphere, using the Schlenk techniques, unless otherwise stated. Deuterated solvents were purchased from Eurisotop and used as received. NMR spectra were recorded in a Bruker AVIIIHD 400 MHz spectrometer equipped with a 5 mm BBFO SmartProbe. ^1^H and ^13^C{^1^H} spectra were referenced with the solvent residual peak. Chemical shift values are reported in ppm and coupling constants (J) in hertz.

Mass spectra were recorded in an Agilent Technologies 6530 Accurate Mass LC–MS QToF.

Stock solutions of complexes **1** and **2** were made up in DMSO and stored at -20 ºC.

### Photophysical characterization

UV–vis absorption spectra in solution were recorded in an Agilent Varian Cary 50 or Agilent Varian Cary 5000 spectrometer using quartz cuvettes of 1 cm pathlength. Luminescence spectra were recorded in a Horiba Jobin Yvon Fluoromax-4 spectrofluorometer. Lifetimes were determined by time-correlated single photon counting (TCSPC) method, using Edinburgh Instrument Mini-tau spectrometer and following excitation with a pulsed laser diode at 405 nm, 75 ps. Lifetime values were determined through exponential fitting of the collected data. Quantum yields were determined by the indirect method, using [Ru(bpy)_3_]Cl_2_ as the standard (0.029 in aerated acetonitrile) [38]. Absorption and emission spectra (at least 6 points) were recorded for increasing concentrations of the samples. The linear fitting from the integrated emission intensity vs. absorption graph, provided the gradient data from the slope. Finally, the quantum yield was calculated using the next equation,

$${\Phi }_{X}= {\Phi }_{ST}\left(\frac{{Grad}_{X}}{{Grad}_{ST}}\right)\left(\frac{{\eta }_{ST}^{2}}{{\eta }_{X}^{2}}\right)$$

where X stands for the complex and ST for the reference or standard, Φ for the quantum yield, Grad for the gradient and η for the refraction index of the solvent. If the solvent of the compound and the reference is the same, the equation is simplified. Quartz cuvettes modified with a young connection tap of 1 cm pathlength were used for deaerated measurements under argon, after three freeze–pump–thaw cycles. pH measurements were performed on a Hanna Instruments 123 Microprocessor pH meter, in a pH range from 2 to 12, using HCl or NaOH solutions to adjust pH.

### (Photo)stability measurements

Stability measurements were performed in aqueous solutions with 2% of DMSO (10^–5^ M for **1** and 2 × 10^–5^ M for **2**) in the dark and after light irradiation with a 405 nm LED diode (Thorlabs M405L4, 20 mW/cm^2^), and tracked by UV–vis absorption. The dark stability was also tracked for **2** (2 × 10^–5^ M) in PBS and white cell media. The photostability experiment for **2** (2 × 10^–5^ M, 70:30 (v/v) DMSO-d_6_/D_2_O) was tracked by ^1^H-NMR in a Bruker 500 MHz spectrometer.

### Cyclic voltammetry

CV experiments were performed using an Autolab 100 potentiostat, with a glassy carbon working electrode, a platinum wire counter electrode and Ag/AgCl (0.1 mol dm^−3^) reference electrode. Solutions of the complexes 10^–3^ M were prepared in CH_2_Cl_2_ using 0.4 M of [NBu_4_][PF_6_] as the supporting electrolyte and were bubbled for at least 15 min with N_2_ to remove any traces of oxygen prior to run the measurements. The last run was recorded with Fc to reference according to the Fc/Fc^+^ couple.

### X-ray diffraction

Crystal structures were solved in a Bruker AXS Venture apparatus. Single crystals of **1** suitable for diffraction were obtained from the slow evaporation of a dichloromethane solution. Crystals were mounted in fomblin oil on a MiTiGen microloop and cooled in a stream of cold N_2_. The structure has been deposited in the CCDC 2284133.

### Singlet oxygen quantum yield

The quantum efficiency of singlet oxygen (^1^O_2_) production was determined by time-resolved near-IR emission at 1270 nm upon laser excitation. An optically matched solution of perinaphthenone was used as the reference with known quantum yield (Φ_s_ = 1.0 in CH_3_CN [41]). The kinetic traces were recorded at a set of different energies of 355-nm, 12-ns output of a LOTIS TII Nd:Yag laser (25, 50, 75 and 100 µJ) for both the sample and perinaphthenone solutions. The amplitude of the emission signal at time = 0 was plotted as a function of excitation energy, and the yield determined by comparing the slopes of such linear dependencies for the sample studied vs. that of perinaphthenone.

### Transient absorption

Femtosecond Transient Absorption (TA) spectroscopy was performed at the Lord Porter Laser Laboratory, University of Sheffield. A Ti:Sapphire regenerative amplifier (Spitfire ACE PA-40, Spectra-Physics) provided 800 nm pulses (40 fs FWHM, 10 kHz, 1.2 mJ). 400 nm pulses for excitation were generated by doubling a portion of the 800 nm output in a β-barium borate crystal within a commercially available doubler/tripler (TimePlate, Photop Technologies). White light supercontinuum probe pulses in the range 340–790 nm were generated with a CaF_2_ crystal (continuously displaced to avoid damage). Detection was achieved using a commercial transient absorption spectrometer (Helios, Ultrafast Systems) using a 2048-pixel CMOS sensor for UV–Vis detection. The relative polarisation of the pump and probe was set to magic angle (54.7˚) for anisotropy-free measurements. Samples were held in 2 mm path length quartz cells in CH_2_Cl_2_ (absorption of 0.4 at the excitation wavelength) and were stirred during experiments.

### Cell line and culture conditions

EJ cells were obtained from ATTC. The cells were maintained in DMEM (Dulbecco’s modified Eagle’s medium, Gibco) with 10% of FBS (foetal bovine serum). Cells were cultured in a humidified incubator at 37 ºC under 5% CO_2_. Cells were regularly tested for mycoplasma.

### Cytotoxicity and phototoxicity

Cytotoxicity and phototoxicity were determined by clonogenic survival assays. For dark toxicity, ells were plated into 6-well plates (200 or 400 cells/well) and allowed to grow overnight. The drugs (stock solutions in DMSO) were added alongside DMSO alone as a vehicle control. The DMSO concentration in the final solutions never exceed 2%. The cells were then incubated for 2 h before removal and replacement of the media. After 7 days, colonies were stained with methylene blue solution (3%, 70:30 ethanol/water) and the colonies counted with a colony counter. For phototoxicity, cells were plated into 12-well plates and allowed to grow overnight. Cells were exposed to drug and incubated for 2 h before being trypsonized and resuspended in phenol-red free media. The cell suspensions were transferred to soda-lime glass vials and preserved on ice. Dark control suspensions were also prepared for each condition. The solutions were irradiated for 3 min with a blue LED diode (Thorlabs, λ = 405 or 455 nm, 20 mW/cm^2^). Each condition was then replated into 6 well dishes at 300 and 600 cells and left for 7 days to form colonies which were then stained and counted. All the experiments were performed in triplicates (at least) and on three separate occasions.

### Dose dependent cell uptake studies

Cells were plated onto glass coverslips in 6 well dishes and incubated overnight. Cells were incubated with 10 μM and 30 μM **1** for 2 h or treated with DMSO control (final DMSO amount 0.2%). Following this, the coverslips were washed with PBS (3 × 1 mL/well) and cells fixed with PFA solution (3%, PBS, 1 mL/well, 10 min, RT). Coverslips were again washed (PBS, 3 × 1 mL/well) before being mounted onto glass slides (ProLong™ Antifade Gold Mountant, 1 drop).

### Imaging

Cell imaging was performed using a Nikon Widefield Live Cell Dual Cam microscope on fixed and mounted coverslips. Excitation was performed using a SpectraX LED source and the emission was passed through a relevant filter before image capture. For each condition, a brightfield and fluorescence image was taken, and a merge presented. Each image was captured as a 3D z-stack before being deconvoluted using a Richardson-Lucy algorithm to reduce out of focus fluorescence.

### Co-localization in mitochondria

EJ cells were plated onto sterilised glass coverslips in a 6-well dish and incubated overnight. Following this, cells were exposed to **1** (30 μM, 2 h) and then fixed in PFA (4% in PBS, 10 min). Cells were then permeabilised with Triton X-100 (0.2% in PBS, 10 min), blocked with BSA (2% in PBS, 1 h) before being exposed to a monoclonal antibody to a mitochondria exclusive protein, cytochrome c oxidase (1:200 in PBS, overnight 4 °C, Abcam, ab133504). The next day, cells were exposed to Alexa Fluor™ 488 anti-rabbit secondary antibody (1:500 in PBS, 1 h) before being mounted onto glass slides (Prolong Gold Antifade). Z-stacked images were then captured with the separate emissions of **1** (red) and anti-cytochrome c oxidase (green) collected per stack. Images were deconvoluted using the Lucy-Richardson algorithm and rendered into a single frame. Pixel overlap and hence co-localisation between the merged emission images of **1** and anti-cytochrome c oxidase were quantified using the Coloc2 program in the image processing software ImageJ.

## Conclusions

We have reported two Ir(III) photosensitizers bearing two cyclometallating ligands, thienyl-pyridine (thpy), and either two mono- or one bidentate imidazole-based ligands, namely 2-(2’-benzothiazolyl)-benzimidazole in **1** and imidazole in **2**. As expected, **1** is photostable, whilst **2** is not. Both complexes absorb visible light, are brightly emissive, and generate singlet oxygen upon irradiation with high yields (40% for **1** and 82% for **2**). The lowest excited state in **2**, without the diimine ligand, is of a pure ^3^IL (intra-thienyl-pyridine) nature, as is indicated by its long lifetime (2 microseconds in deoxygenated solution), and clear vibrational progression in the emission spectrum. However, complex **2** was found to be toxic in the dark, potentially due to dissociation of imidazole ligand. It is thus not suitable for PDT but could form a basis for development of a future drug working through release of imidazole, joining a large family of agents based on imidazole scaffolds [46].

Complex **1** carries a bidentate, diimine ligand, and possesses a manifold of lowest excited states of a mixed ^3^IL/^3^MLCT origin, as is evidenced by the shape of its emission spectrum. **1** was shown to be a good PS agent, non-toxic in dark conditions, and with a PI > 250 after irradiation at 405 nm and PI > 150 at 455 nm, after short irradiation time and at low fluences. Overall, this study contributes to the development of metal complexes for therapies, and offers a new photosensitizer, **1**, which works under visible light, in a set of cancer-related cell lines, at low fluences and low dose of light.

### Supplementary Information

Below is the link to the electronic supplementary material.

Supplementary file1 (PDF 1354 KB)

Supplementary file1 (CIF 776 KB)

Supplementary file3 (PDF 89 KB)

## Data Availability

Crystallographic data for the structure reported in this article has been deposited at the Cambridge Crystallographic Data Centre, under deposition number CCDC 2284133 (1). Copies of the data can be obtained free of charge via https://www.ccdc.cam.ac.uk/structures/. The authors declare that the data supporting the findings of this study are available within the paper and its Supplementary Information files. Should any raw data files be needed in another format they are available from the corresponding authors upon reasonable request.
