# Supplementary material for: Phototoxicity of cyclometallated Ir(III) complexes bearing a thio-bis-benzimidazole ligand, and its monodentate analogue, as potential PDT photosensitisers in cancer cell killing
Source: J Biol Inorg Chem. 2024 Jan 6;29(1):113–25. doi: 10.1007/s00775-023-02031-z (PMC11001735; doi:10.1007/s00775-023-02031-z)
Supplement: Supplementary file 1 — Supplementary file1 (PDF 1354 KB) [file 775_2023_2031_MOESM1_ESM.pdf]

Supplementary information

Content

Characterization (^{1}H-NMR, ^{13}C-NMR, ESI-MS) 2
Complex 1 2
Complex 2 5
X-Ray Diffraction 8
Photophysical Properties 10
Photostability 11
pH titration 13

Characterization (^{1}H-NMR, ^{13}C-NMR, ESI-MS)

<u>Complex 1</u>

^{1}H-NMR

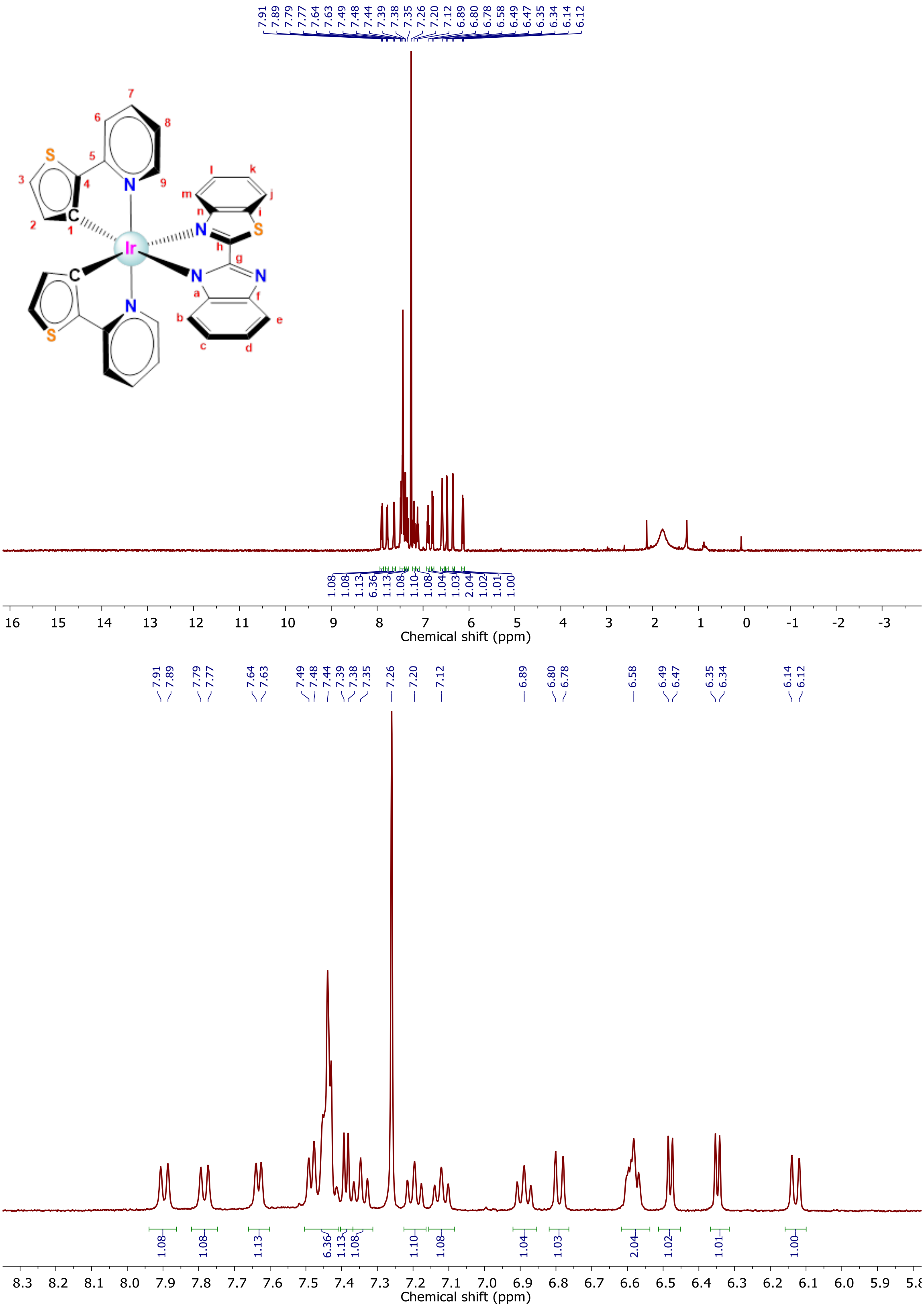

Figure S1. ^{1}H-NMR spectrum of **1** in $CDCl_3$ (full range - up, aromatic region – down).

^{13}C-NMR

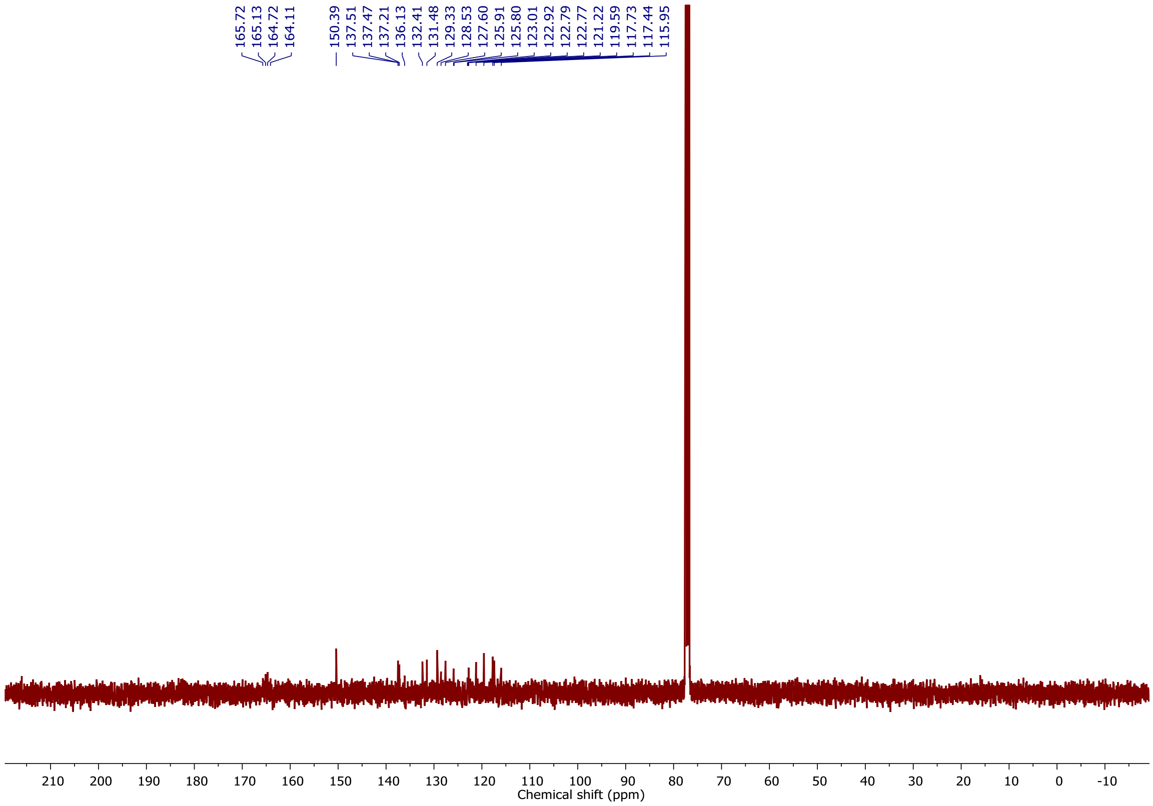

Figure S2. ^{13}C-NMR spectrum of **1** in $CDCl_3$.

ESI-MS

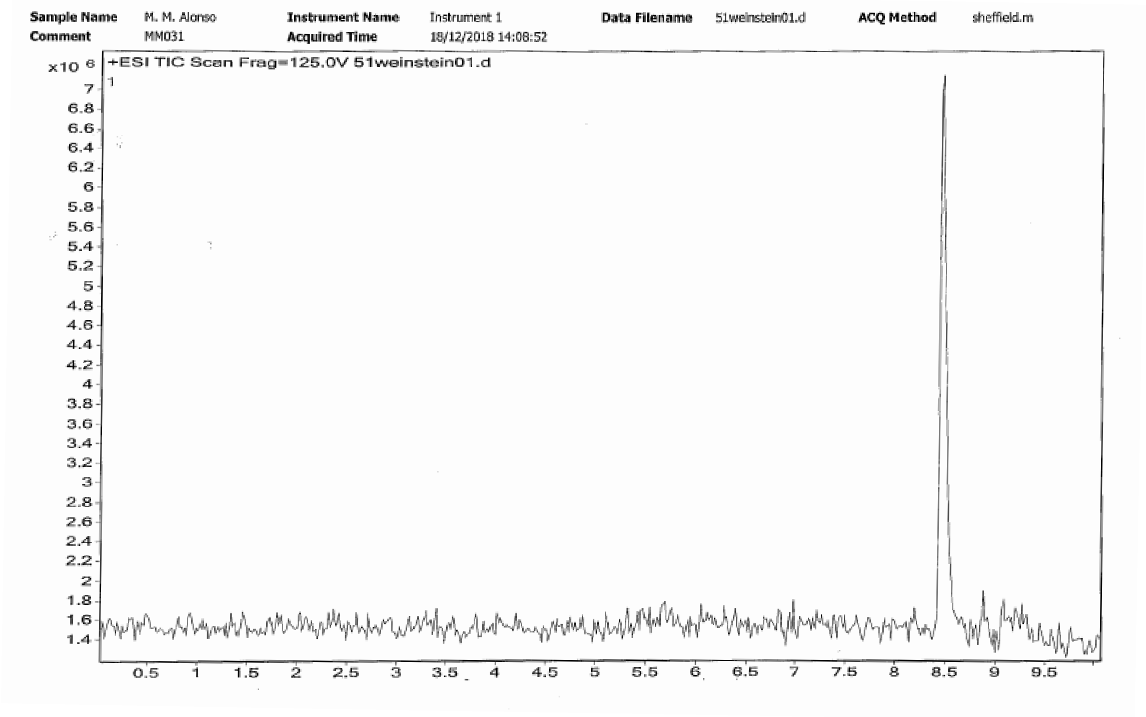

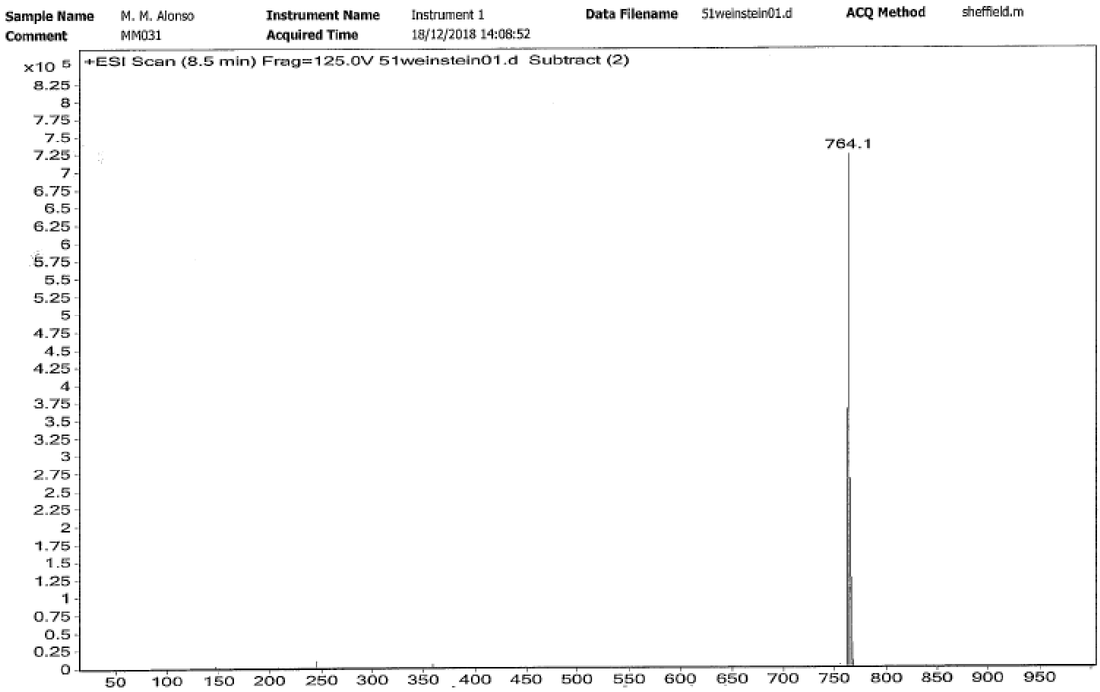

Figure S3. ESI-MS spectrum of **1**.

Complex 2

^{1}H-NMR

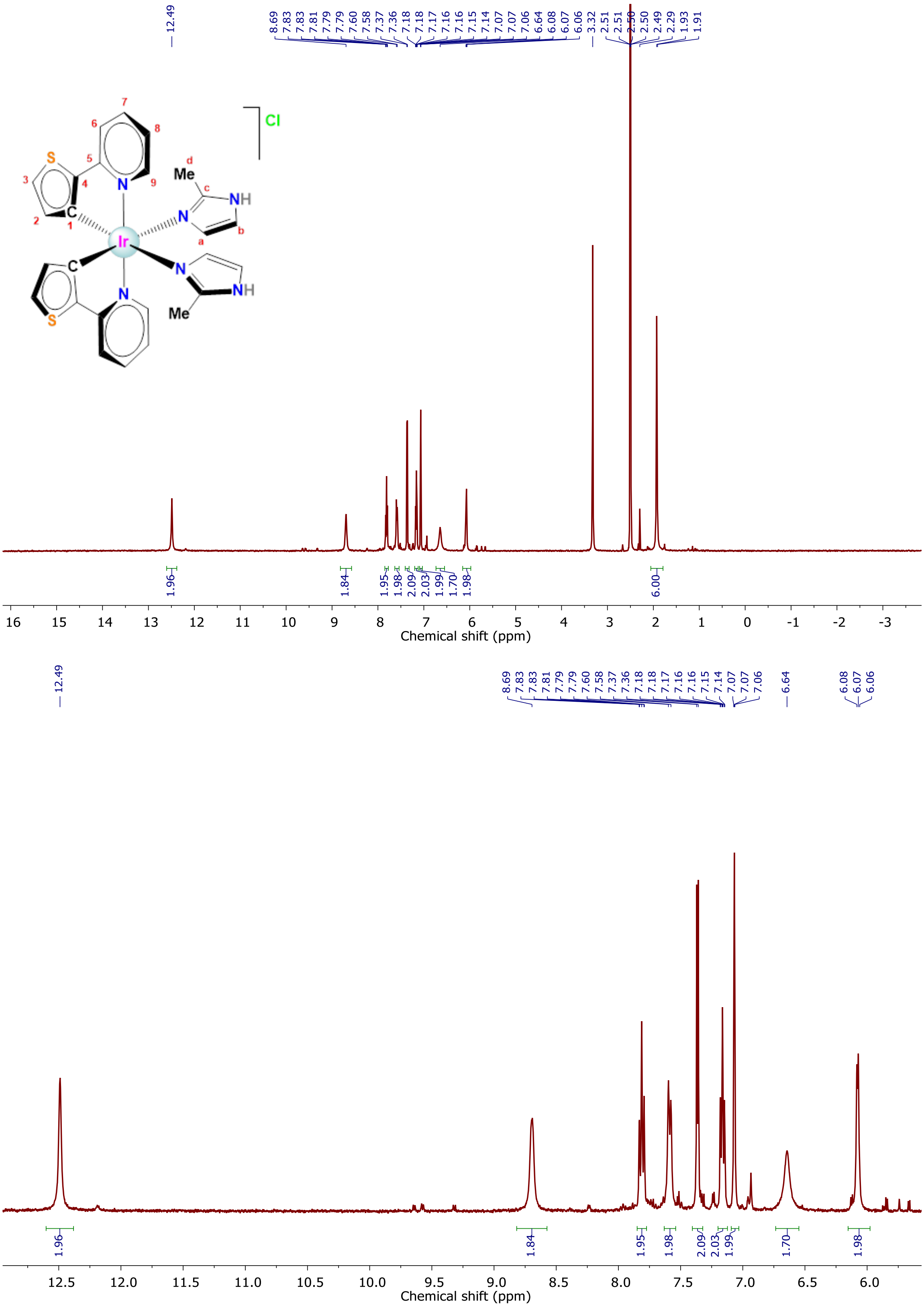

Figure S4. ^{1}H-NMR spectrum of **2** in DMSO-d_6 (full range - up, aromatic region – down).

^{13}C-NMR

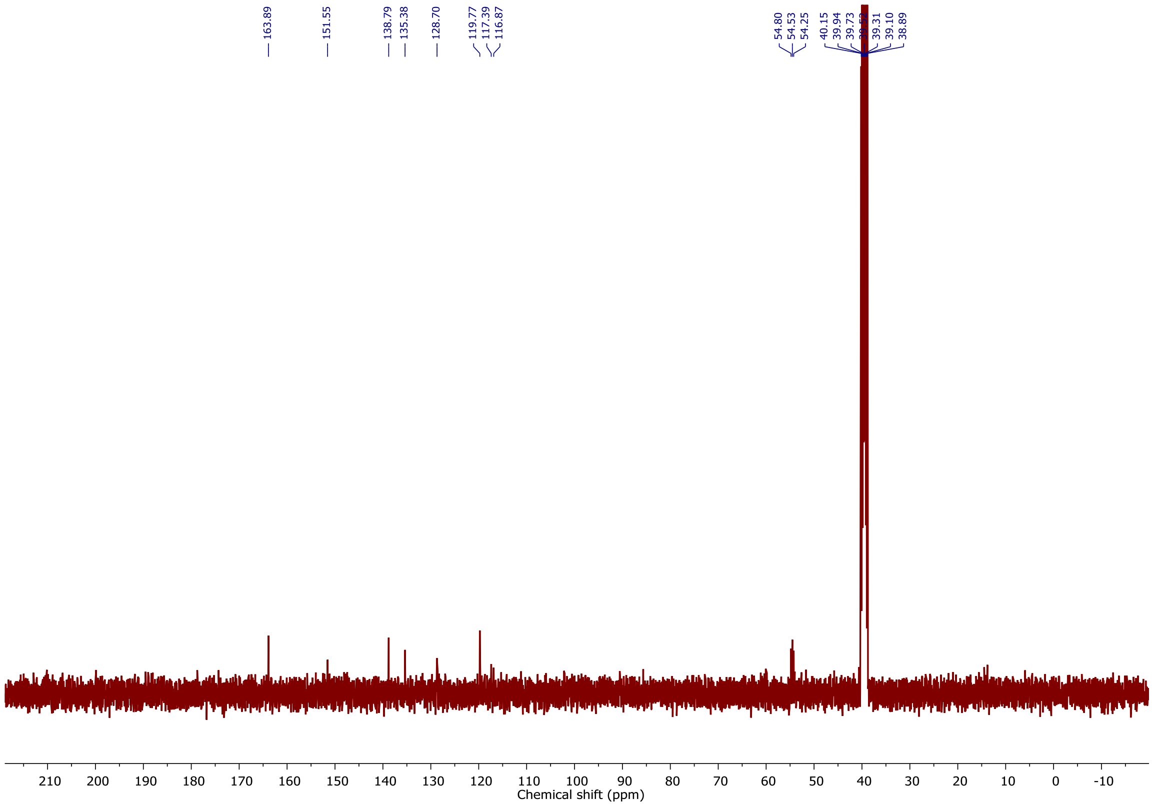

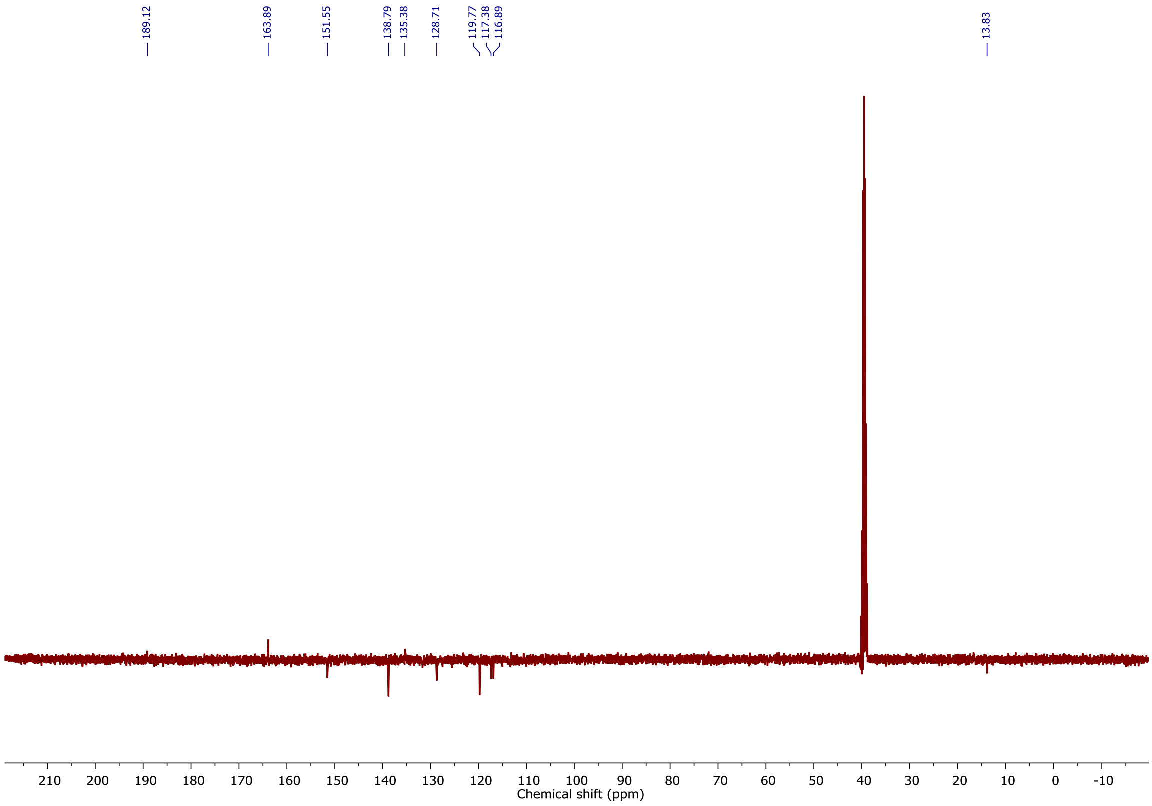

Figure S5. ^{13}C-NMR spectrum (up) and DEPTQ (down) of **1** in $CDCl_3$.

ESI-MS

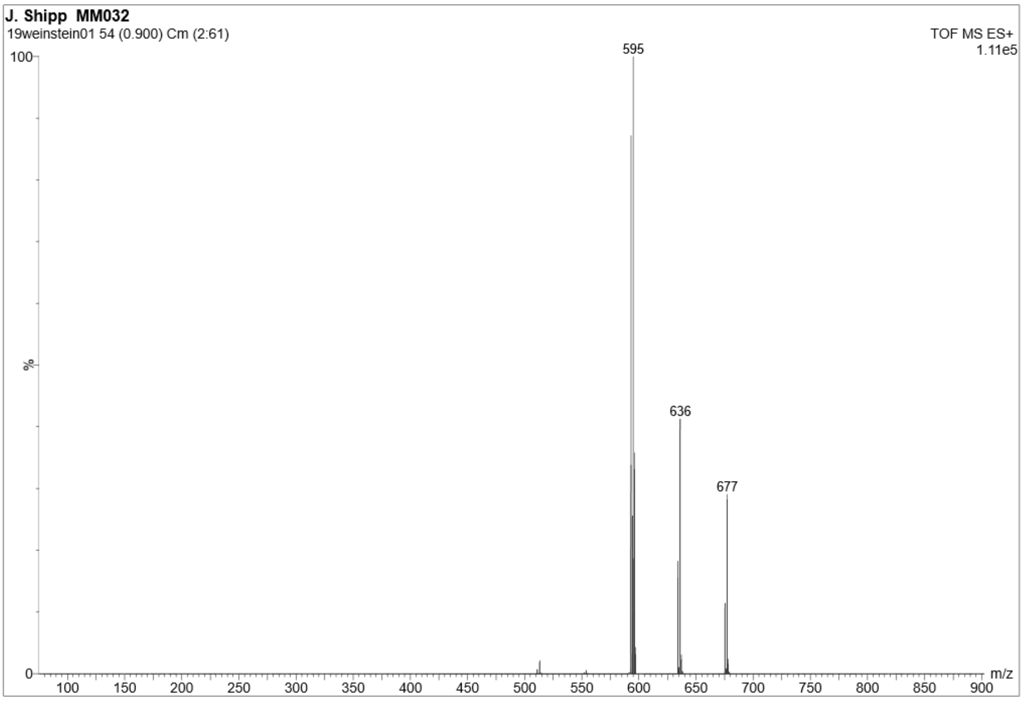

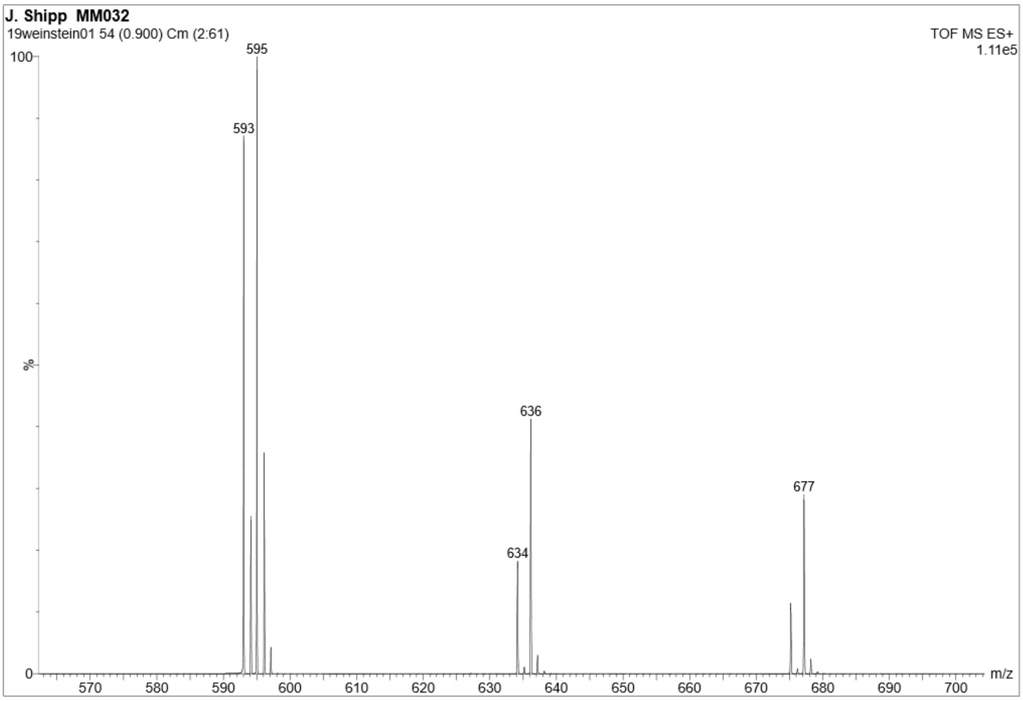

Figure S6. ESI-MS spectrum of **1**.

X-Ray Diffraction

Table S1. Crystal data and structure refinement for **1**.

**Empirical formula**	$C_{67}H_{46}Cl_6Ir_2N_{10}S_6$
**Formula weight**	1780.60
**Temperature/K**	100
**Crystal system**	orthorhombic
**Space group**	$Pca2_1$
**a/Å**	17.948(2)
**b/Å**	9.2055(11)
**c/Å**	39.053(5)
**α/°**	90
**β/°**	90
**γ/°**	90
**Volume/Å^3**	6452.4(13)
**Z**	4
**ρ_{calc}g/cm^3**	1.833
**μ/mm^{-1}**	4.616
**F(000)**	3480.0
**Crystal size/mm^3**	0.1 × 0.1 × 0.02
**Radiation**	MoKα (λ = 0.71073)
**2Θ range for data collection/°**	2.498 to 49.45
**Index ranges**	-19 ≤ h ≤ 21, -9 ≤ k ≤ 10, -45 ≤ l ≤ 45
**Reflections collected**	24374
**Independent reflections**	10936 [R_{int} = 0.0950, R_{sigma} = 0.1500]
**Data/restraints/parameters**	10936/872/503
**Goodness-of-fit on F^2**	1.032
**Final R indexes [I>=2σ (I)]**	R_1 = 0.0771, wR_2 = 0.1365
**Final R indexes [all data]**	R_1 = 0.1317, wR_2 = 0.1572
**Largest diff. peak/hole / e Å^{-3}**	2.72/-2.20
**Flack parameter**	0.013(13)

Table S2. Selected bond lengths (Å) for **1**.

Bond	d (error) /Å	Bond	d (error) /Å
**Ir1A-N1A**	2.15 (3)	**Ir1B-N1B**	2.15 (3)
**Ir1A-N2A**	2.09 (3)	**Ir1B-N3B**	2.20 (3)
**Ir1A-N4A**	2.02 (3)	**Ir1B-N4A**	2.04 (3)
**Ir1A-N5A**	2.04 (3)	**Ir1B-N5B**	2.07 (2)
**Ir1A-C15A**	2.02 (3)	**Ir1B-C15B**	1.96 (3)
**Ir1A-C24A**	2.01 (3)	**Ir1B-C24B**	1.98 (3)

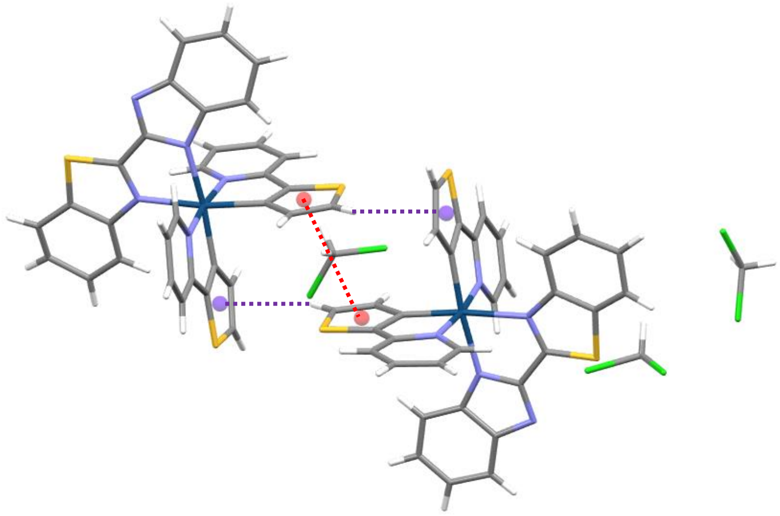

Figure S7. π-π and CH-π stacking interactions involving the thiazole ring of the cyclometallating ligands.

Table S3. Parameters of the π-π stacking interaction of **1**.

Compound	$d_{cent\text{-}cent}$ (Å)	α (°)	$d_{cent\text{-}pl}$ (Å)	β (°)	d_{offset} (Å)
**1**	4.209	1.45	3.554	32.39	2.255
			3.544	32.65	2.271

Table S4. Parameters of the CH-π interaction of **1**.

Bond	$d_{C\text{-}cent}$ (Å)	$d_{H\text{-}cent}$ (Å)	$d_{C\text{-}H}$ (Å)	C-H-cent (°)	H-cent-normal (°)
**C-H(Λ)···π (Δ)**	3.722	2.951	0.953	138.84	163.47
**C-H(Δ)···π (Λ)**	3.685	2.961	0.951	133.95	158.55

Table S5. Hydrogen bond parameters for **1**.

Bond	D···A (Å)	H···A (Å)	D···H (Å)	α (°)
**Cl_2C-H···N(Λ)**	3.179	2.274	0.989	151.59
**C(ring)-H···N(Δ)**	3.401	2.642	0.952	136.97

Photophysical Properties

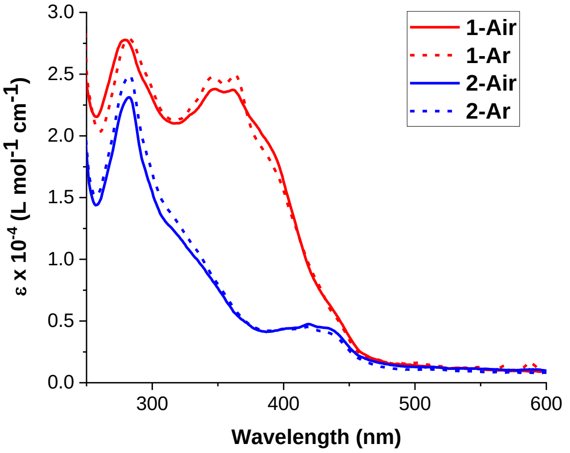

Figure S8. UV-vis absorption spectra of complexes **1** and **2** aerated (solid line) and under argon (dash line) in CH_2Cl_2.

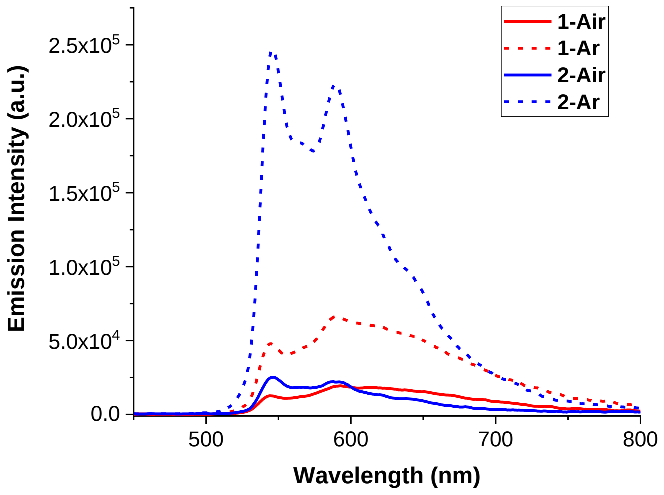

Figure S9. PL emission spectra of complexes **1** (10^{-5} M) and **2** (2×10^{-5} M) aerated (solid line) and under argon (dash line) in CH_2Cl_2.

Photostability

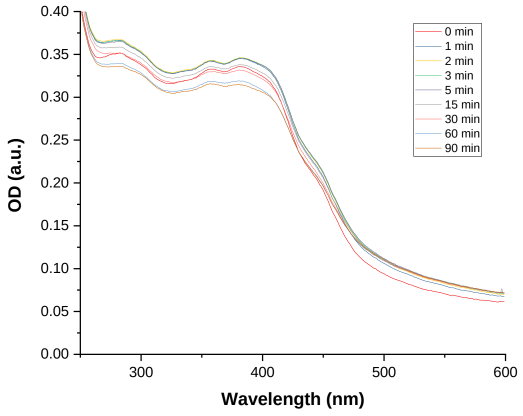

Figure S10. Time-dependent UV-vis spectra of complex **1** in water (1 % DMSO) after irradiation (405 nm, 20 mW).

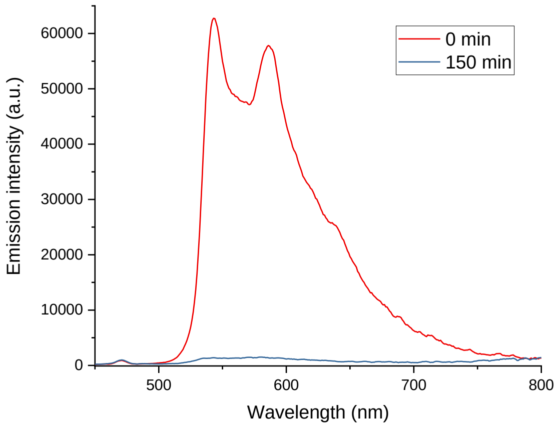

Figure S11. PL spectra of complex **2** in water (2 % DMSO) after 150 min irradiation (405 nm, 20 mW).

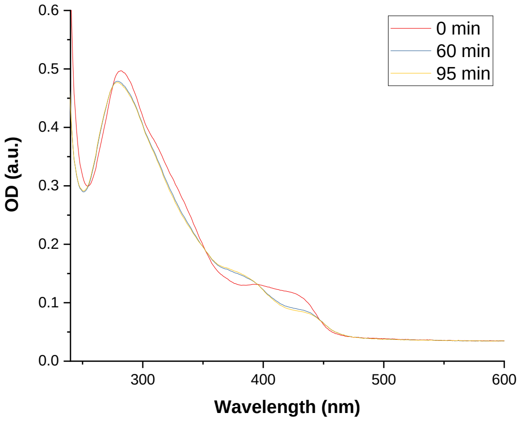

Figure S12. Time-dependent UV-vis spectra of complex **2** in water (2 % DMSO) exposed to ambient light for 95 min.

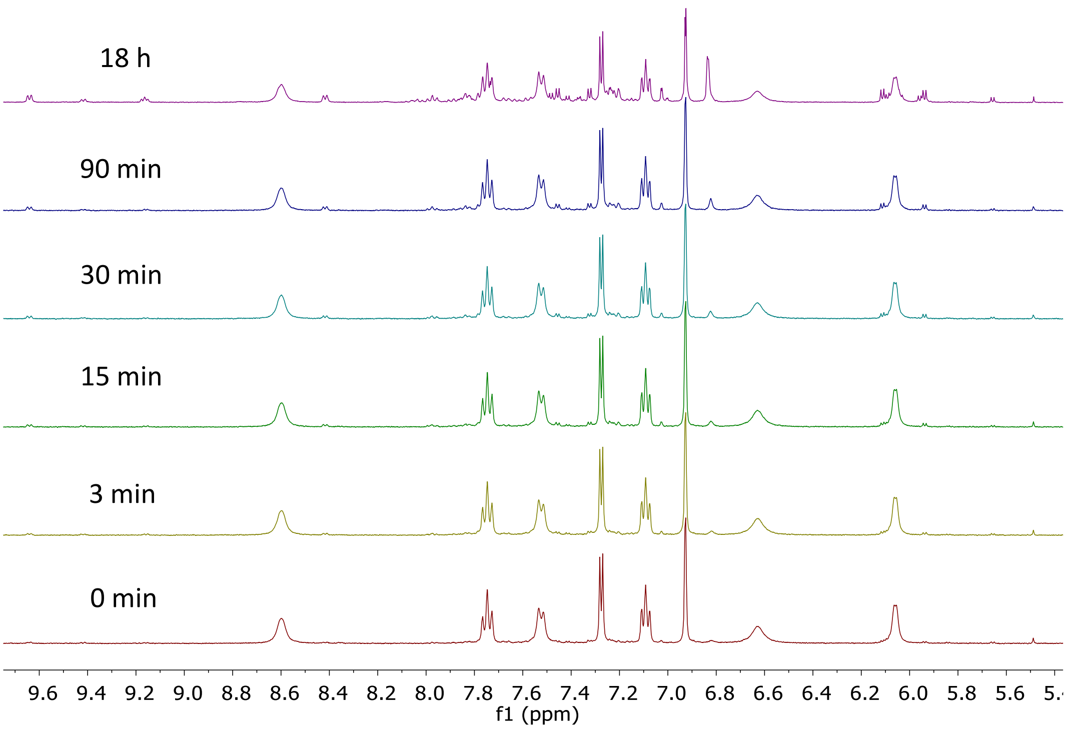

Figure S13. Time-dependent ^{1}H-NMR spectrum of complex **2** (10 mM) in DMSO-d$_6$/D$_2$O (70:30) at different irradiation times (405 nm, 20 mW); aromatic region.

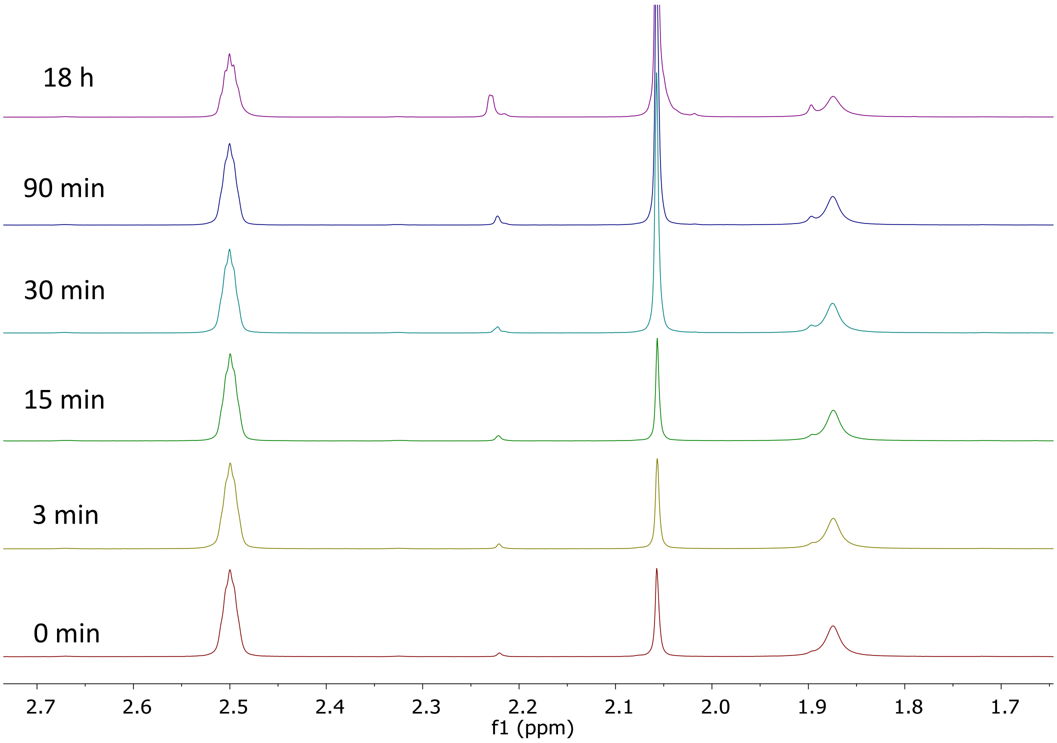

Figure S14. Time-dependent ^{1}H-NMR spectrum of complex **2** (10 mM) in DMSO-d$_6$/D$_2$O (70:30) at different irradiation times (405 nm, 20 mW); aliphatic region.

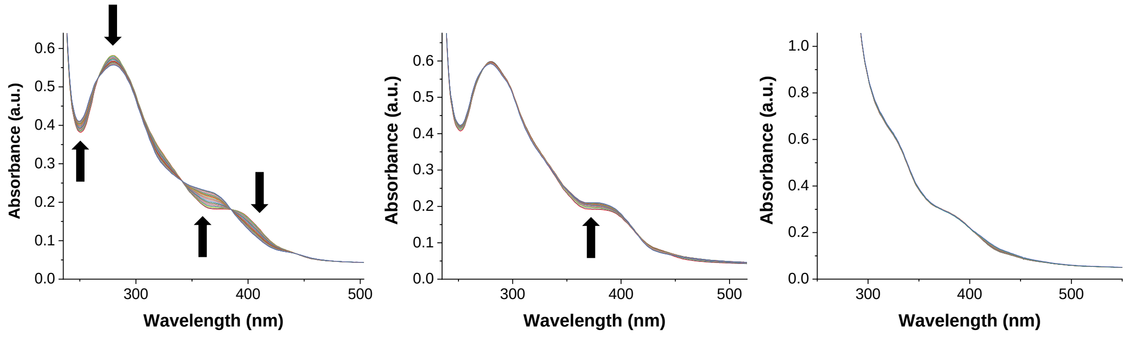

Figure S15. Time-dependent UV-vis spectra of complex **2** in dark conditions in a) water (2 % DMSO), b) PBS and c) white media.

pH titration

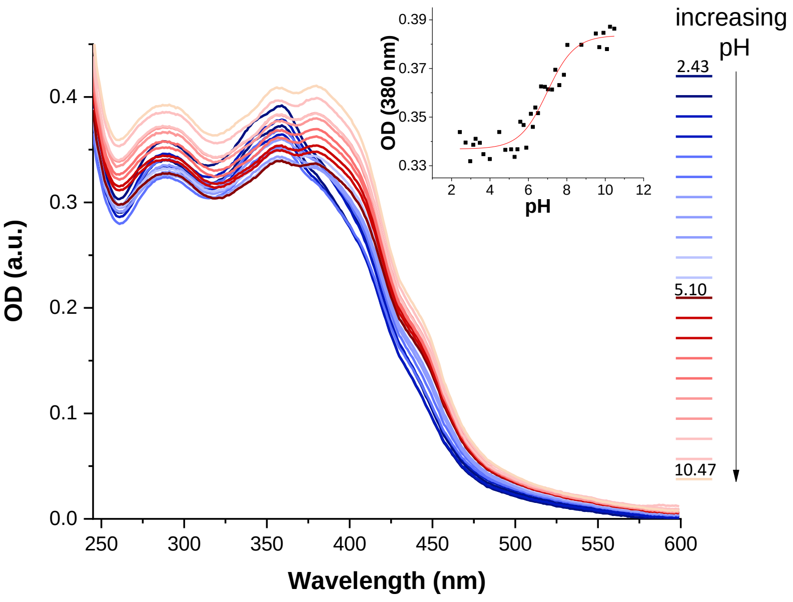

Figure S16. pH titration of **1** in PBS.

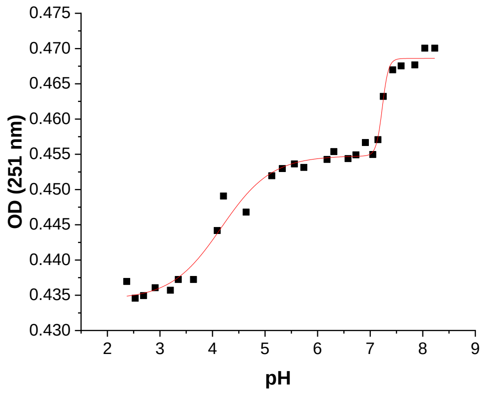

Figure S17. pH titration of **2** in PBS.
