# Supplementary material for: Phototoxicity of cyclometallated Ir(III) complexes bearing a thio-bis-benzimidazole ligand, and its monodentate analogue, as potential PDT photosensitisers in cancer cell killing
Source: J Biol Inorg Chem. 2024 Jan 6;29(1):113–25. doi: 10.1007/s00775-023-02031-z (PMC11001735; doi:10.1007/s00775-023-02031-z)

checkCIF/PLATON report

Structure factors have been supplied for datablock(s) ch1jw279k_0m

THIS REPORT IS FOR GUIDANCE ONLY. IF USED AS PART OF A REVIEW PROCEDURE FOR PUBLICATION, IT SHOULD NOT REPLACE THE EXPERTISE OF AN EXPERIENCED CRYSTALLOGRAPHIC REFEREE.

No syntax errors found. CIF dictionary Interpreting this report

Datablock: ch1jw279k_0m

Bond precision:	C-C = 0.0472 A		Wavelength=0.71073
Cell:	a=17.948(2)	b=9.2055(11)	c=39.053(5)
	alpha=90	beta=90	gamma=90
Temperature:	100 K		

	Calculated	Reported
Volume	6452.4(13)	6452.4(13)
Space group	P c a 21	P c a 21
Hall group	P 2c -2ac	P 2c -2ac
Moiety formula	2(C32 H20 Ir N5 S3), 3(C H2 Cl2)	2(C32 H20 Ir N5 S3), 3(C H2 Cl2)
Sum formula	C67 H46 Cl6 Ir2 N10 S6	C67 H46 Cl6 Ir2 N10 S6
Mr	1780.64	1780.60
Dx,g cm-3	1.833	1.833
Z	4	4
Mu (mm-1)	4.616	4.616
F000	3480.0	3480.0
F000'	3476.04	
h,k,lmax	21,10,45	21,10,45
Nref	11014[5599]	10936
Tmin,Tmax	0.637,0.912	0.516,0.745
Tmin'	0.624	

Correction method= # Reported T Limits: Tmin=0.516 Tmax=0.745
AbsCorr = MULTI-SCAN

Data completeness= 1.95/0.99 Theta(max)= 24.725

R(reflections)= 0.0776(7201) wR2(reflections)= 0.1589(10936)

S = 1.032 Npar= 503

The following ALERTS were generated. Each ALERT has the format
test-name_ALERT_alert-type_alert-level.
Click on the hyperlinks for more details of the test.

Alert level B

```
PLAT342_ALERT_3_B Low Bond Precision on  C-C Bonds ..............    0.04724 Ang.
```

Alert level C

```
THETM01_ALERT_3_C  The value of sine(theta_max)/wavelength is less than 0.590
            Calculated sin(theta_max)/wavelength =    0.5885
PLAT234_ALERT_4_C Large Hirshfeld Difference S2B      --C17B     .        0.19 Ang.
PLAT241_ALERT_2_C High   'MainMol' Ueq as Compared to Neighbors of         N3A Check
PLAT243_ALERT_4_C High   'Solvent' Ueq as Compared to Neighbors of         C1S Check
PLAT243_ALERT_4_C High   'Solvent' Ueq as Compared to Neighbors of         C3S Check
PLAT244_ALERT_4_C Low    'Solvent' Ueq as Compared to Neighbors of         C2S Check
PLAT260_ALERT_2_C Large Average Ueq of Residue Including      Cl1S      0.120 Check
PLAT260_ALERT_2_C Large Average Ueq of Residue Including      Cl5S      0.199 Check
PLAT411_ALERT_2_C Short Inter H...H Contact  H5B      ..H3SB     .        2.08 Ang.
                                           1/2+x,2-y,z  =      3_575 Check
PLAT906_ALERT_3_C Large K Value in the Analysis of Variance ......      2.639 Check
PLAT911_ALERT_3_C Missing FCF Refl Between Thmin & STh/L=    0.589           5 Report
PLAT971_ALERT_2_C Check Calcd Resid. Dens.  0.85Ang From Ir1B           2.22 eA-3
PLAT971_ALERT_2_C Check Calcd Resid. Dens.  1.07Ang From Ir1A           2.00 eA-3
PLAT971_ALERT_2_C Check Calcd Resid. Dens.  1.91Ang From C22A           1.95 eA-3
PLAT971_ALERT_2_C Check Calcd Resid. Dens.  1.09Ang From Cl6S           1.90 eA-3
PLAT971_ALERT_2_C Check Calcd Resid. Dens.  1.10Ang From Ir1B           1.82 eA-3
PLAT971_ALERT_2_C Check Calcd Resid. Dens.  1.15Ang From Ir1A           1.69 eA-3
PLAT972_ALERT_2_C Check Calcd Resid. Dens.  2.07Ang From C3A           -2.07 eA-3
PLAT972_ALERT_2_C Check Calcd Resid. Dens.  1.96Ang From C30B          -2.06 eA-3
PLAT972_ALERT_2_C Check Calcd Resid. Dens.  0.88Ang From Ir1B          -2.05 eA-3
PLAT972_ALERT_2_C Check Calcd Resid. Dens.  0.82Ang From Ir1B          -1.84 eA-3
PLAT972_ALERT_2_C Check Calcd Resid. Dens.  0.81Ang From Ir1B          -1.69 eA-3
PLAT972_ALERT_2_C Check Calcd Resid. Dens.  0.87Ang From Ir1A          -1.62 eA-3
PLAT972_ALERT_2_C Check Calcd Resid. Dens.  0.91Ang From N1A           -1.61 eA-3
PLAT972_ALERT_2_C Check Calcd Resid. Dens.  0.79Ang From Ir1A          -1.59 eA-3
PLAT972_ALERT_2_C Check Calcd Resid. Dens.  0.87Ang From Ir1A          -1.55 eA-3
PLAT972_ALERT_2_C Check Calcd Resid. Dens.  1.85Ang From Cl3S          -1.54 eA-3
PLAT977_ALERT_2_C Check Negative Difference Density on H2SB      .      -0.34 eA-3
```

Alert level G

```
PLAT002_ALERT_2_G Number of Distance or Angle Restraints on AtSite          3 Note
PLAT003_ALERT_2_G Number of Uiso or Uij Restrained non-H Atoms ...          3 Report
PLAT083_ALERT_2_G SHELXL Second Parameter in WGHT  Unusually Large     139.15 Why ?
PLAT171_ALERT_4_G The CIF-Embedded .res File Contains EADP Records          7 Report
PLAT176_ALERT_4_G The CIF-Embedded .res File Contains SADI Records          1 Report
PLAT177_ALERT_4_G The CIF-Embedded .res File Contains DELU Records          1 Report
PLAT178_ALERT_4_G The CIF-Embedded .res File Contains SIMU Records          1 Report
PLAT187_ALERT_4_G The CIF-Embedded .res File Contains RIGU Records          1 Report
PLAT335_ALERT_2_G Check Large C6 Ring C-C Range C1A      -C6A          0.18 Ang.
PLAT344_ALERT_2_G Unusual sp3       Angle Range in Solvent/Ion for        C3S Check
PLAT720_ALERT_4_G Number of Unusual/Non-Standard Labels ..........          6 Note
PLAT790_ALERT_4_G Centre of Gravity not Within Unit Cell: Resd.  #          3 Note
```

```
              C H2 Cl2
PLAT790_ALERT_4_G Centre of Gravity not Within Unit Cell: Resd.  #          4 Note
              C H2 Cl2
PLAT860_ALERT_3_G Number of Least-Squares Restraints ............         798 Note
PLAT909_ALERT_3_G Percentage of I>2sig(I) Data at Theta(Max) Still        37% Note
PLAT910_ALERT_3_G Missing # of FCF Reflection(s) Below Theta(Min).          1 Note
PLAT941_ALERT_3_G Average HKL Measurement Multiplicity ..........         4.1 Low
PLAT978_ALERT_2_G Number C-C Bonds with Positive Residual Density.          0 Info
```

```
   0 ALERT level A = Most likely a serious problem - resolve or explain
   1 ALERT level B = A potentially serious problem, consider carefully
  28 ALERT level C = Check. Ensure it is not caused by an omission or oversight
  18 ALERT level G = General information/check it is not something unexpected

   0 ALERT type 1 CIF construction/syntax error, inconsistent or missing data
  27 ALERT type 2 Indicator that the structure model may be wrong or deficient
   8 ALERT type 3 Indicator that the structure quality may be low
  12 ALERT type 4 Improvement, methodology, query or suggestion
   0 ALERT type 5 Informative message, check
```

It is advisable to attempt to resolve as many as possible of the alerts in all categories. Often the minor alerts point to easily fixed oversights, errors and omissions in your CIF or refinement strategy, so attention to these fine details can be worthwhile. In order to resolve some of the more serious problems it may be necessary to carry out additional measurements or structure refinements. However, the purpose of your study may justify the reported deviations and the more serious of these should normally be commented upon in the discussion or experimental section of a paper or in the "special_details" fields of the CIF. checkCIF was carefully designed to identify outliers and unusual parameters, but every test has its limitations and alerts that are not important in a particular case may appear. Conversely, the absence of alerts does not guarantee there are no aspects of the results needing attention. It is up to the individual to critically assess their own results and, if necessary, seek expert advice.

Publication of your CIF in IUCr journals

A basic structural check has been run on your CIF. These basic checks will be run on all CIFs submitted for publication in IUCr journals (*Acta Crystallographica*, *Journal of Applied Crystallography*, *Journal of Synchrotron Radiation*); however, if you intend to submit to *Acta Crystallographica Section C* or *E* or *IUCrData*, you should make sure that full publication checks are run on the final version of your CIF prior to submission.

Publication of your CIF in other journals

Please refer to the *Notes for Authors* of the relevant journal for any special instructions relating to CIF submission.

PLATON version of 06/07/2023; check.def file version of 30/06/2023

Datablock ch1jw279k_0m - ellipsoid plot

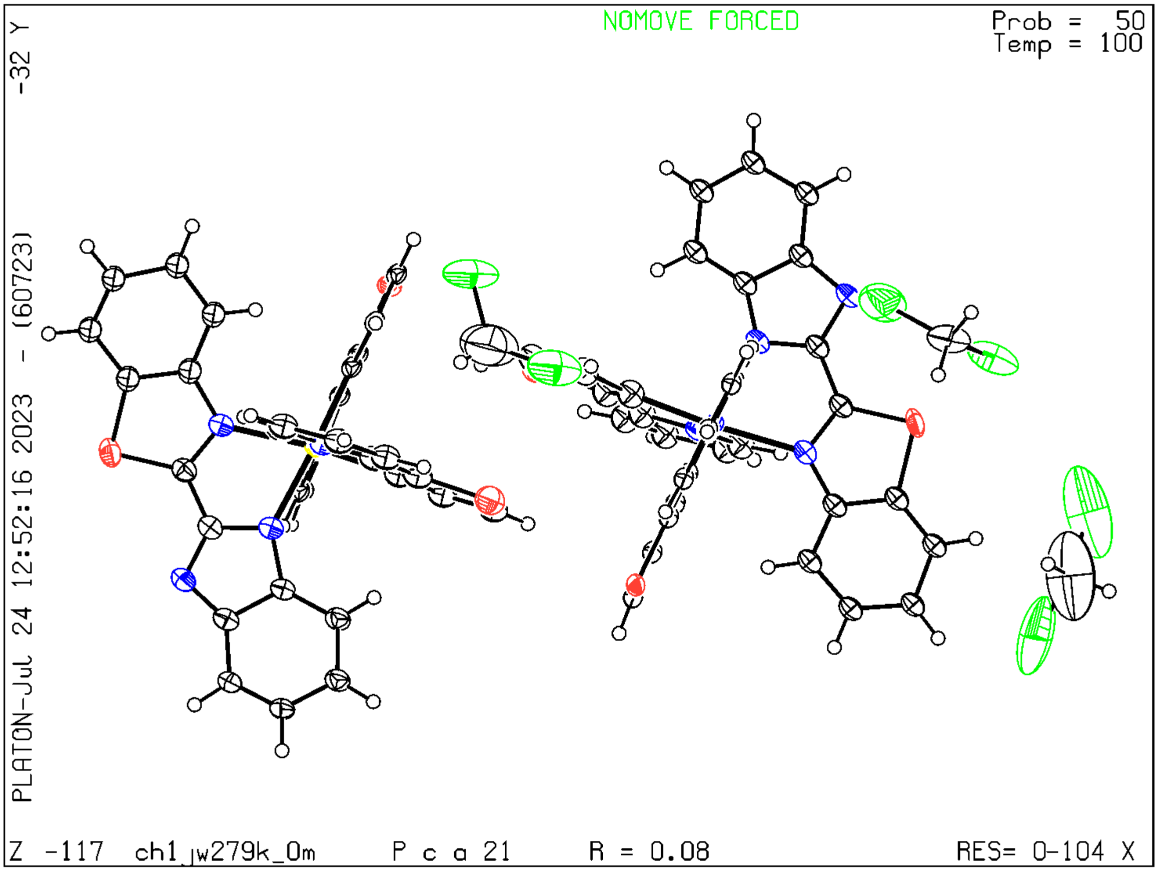

Supplement: Supplementary file 3 — Supplementary file3 (PDF 89 KB) [file 775_2023_2031_MOESM3_ESM.pdf]
